# Enhancing Specificity, Precision, Accessibility, Flexibility, and Safety to Overcome Traditional CRISPR/Cas Editing Challenges and Shape Future Innovations

**DOI:** 10.1002/advs.202416331

**Published:** 2025-06-23

**Authors:** Muna Alariqi, Mohamed Ramadan, Lu Yu, Fengjiao Hui, Amjad Hussain, Xiaofeng Zhou, Yu Yu, Xianlong Zhang, Shuangxia Jin

**Affiliations:** ^1^ Hubei Hongshan Laboratory National Key Laboratory of Crop Genetic Improvement Huazhong Agricultural University Wuhan 430070 China; ^2^ Department of Crops Science and Genetic Improvement Faculty of Agriculture Food and Environment Sana'a University Sana'a 19065 Yemen; ^3^ Department of Plant Genetic Resources Division of Ecology and Dry Land Agriculture Desert Research Center Cairo Egypt; ^4^ Cotton Research Institute Xinjiang Academy of Agriculture and Reclamation Science Shihezi Xinjiang 832000 China

**Keywords:** CRISPR/Cas, delivery systems, double‐stranded breaks, genome editing, HDR, off‐target

## Abstract

Derived from the bacterial immune system, CRISPR/Cas9 induces DSBs at specific DNA sequences, which are repaired by the cell's endogenous mechanisms, leading to gene insertions, deletions, or substitutions. Despite its transformative potential, several challenges remain in optimizing of CRISPR/Cas systems, including off‐target effects, delivery methods, PAM restrictions, and the limitations of traditional editing approaches. This review focuses on the interplay between these challenges and their contributions to gene editing precision, specificity, accessibility, flexibility, and safety. How reducing off‐target effects enhances specificity and safety is explored, while discussing the role of HDR‐based editing in achieving precise gene modifications, alongside alternative methods such as base editing and prime editing. Improved delivery mechanisms are examined for their impact on accessibility and efficiency, while the reduction of PAM restrictions is highlighted for its contributions to flexibility. Lastly, emerging cleavage‐free editing technologies are evaluated as they relate to safety and accessibility. This focused review aims to clarify the connections among these aspects and outline future research directions for advancing CRISPR‐based applications.

## Graphical abstract

CRISPR/Cas9, while transformative, faces challenges in specificity, precision, delivery, accessibility, flexibility, and safety. This review addresses these limitations by highlighting strategies to reduce off‐target effects, exploring HDR‐based and alternative editing approaches, and evaluating advanced delivery mechanisms. Base and prime editors offer safer, precise alternatives, broadening applications and enhancing therapeutic potential. It further clarifies these interconnections and outlines future research directions.

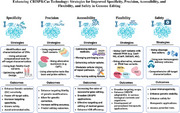

## 1 Background

The term “genome editing” refers to those technologies that enable changes in the building blocks of the DNA, resulting in mutagenesis at any locus in the genome. Unlike early genome editing techniques, modern genome editing tools, led by the Clustered Regularly Interspaced Short Palindromic Repeats (CRISPR)‐derived RNA technology, can induce site‐directed mutagenesis, including insertion, deletion, or substitution, mainly by generating double‐stranded breaks (DSBs) at a specific position in the DNA via engineered sequence‐specific nucleases. Various CRISPR‐derived Cas nucleases have been identified, enabling DNA targeting in different living organisms. Interestingly, by taking advantages of the high targeting specificity of CRISPR technology, the functionality of CRISPR tools has been extended beyond gene disruption (knock‐out) and went further to achieve gene regulation (knock‐up, knockdown, and epigenetic modification), precise gene insertion (knock‐in), and gene correction.^[^ ^1^ ^]^ This revolutionary technology has transformed various sectors during the last decade, including agriculture, healthcare, and beyond, enabling precise genetic modifications that were previously impractical. In agriculture, CRISPR has facilitated remarkable advances, leading to the development of traits that enhance both productivity and sustainability. For instance, it has produced slick‐coat cattle that contribute to better heat stress tolerance, resistant pigs to porcine reproductive and respiratory syndrome virus,^[^ ^2^ ^]^ red sea bream with high skeletal muscle growth,^[^ ^3^, ^4^, ^5^ ^]^ as well as tiger pufferfish with increased appetites.^[^ ^6^ ^]^ Additionally, innovations such as high‐oleic soybeans,^[^ ^7^ ^]^ tomatoes enriched with gamma‐aminobutyric acid (GABA),^[^ ^8^ ^]^ high‐ yield and waxy maize,^[^ ^9^, ^10^ ^]^ and bananas that resist browning^[^ ^11^ ^]^ are already making significant impacts in the market. As of now, CRISPR boasts dozens of approved and experimental applications, extending its influence well beyond agriculture. Notably, it has the potential to make cows safer for the planet^[^ ^12^ ^]^ while inspiring innovations in human health, such as treatments for Alzheimer's disease.^[^ ^13^ ^]^ Perhaps its most celebrated achievement is the successful treatment of sickle cell anemia. In 2022, the FDA approved Casgevy, a groundbreaking CRISPR therapy for this disease, marking a historic milestone in the journey from academic research to clinical application.^[^ ^14^ ^]^ This rapid evolution underscores not only the transformative potential of CRISPR technology but also its capacity to address some of the most pressing challenges confronting humanity today.

Various CRISPR‐derived genome editing systems have been identified and classified based on: i) the corresponding signature Cas protein (e.g., Cas9, Cas12, Cas13, etc.) and ii) protospacer‐adjacent motif (PAM) requirement. CRISPR systems are divided into two classes (I and II), six types (I – VI), and several sub‐types. Class I systems include types I, III, and IV harboring multi‐Cas effector proteins, while Class II systems consist of types II, V, and VI with a single effector protein.^[^ ^15^, ^16^, ^17^ ^]^ Although several CRISPR/Cas variants for genome editing have been proposed, members of Class 2 have attracted researchers’ attention. The CRISPR/Cas9 system from *Streptococcus pyogenes* (SpCas9), in particular, has become predominant over the other CRISPR/Cas variants. The employment of CRISPR/Cas9 technology has made gene manipulation easier, faster, and cheaper, resulting in a significant number of studies aiming to tailor efficient gene modification referring to CRISPR technology. Despite the transformative revolution in the applied science by CRISPR platforms and contrary to initial predictions, CRISPR technology has unveiled more limitations than expected (i.e., off‐target effects, delivery methods, PAM restrictions, and the induction of DSBs etc.), lessening editing efficiency or, in some cases, preventing editing altogether. These challenges have paved the way for novel discoveries that harness the fundamental capability of the CRISPR toolbox to address the existing limitations. The following section of this review sheds light on these limitations, highlights the significant discoveries made over the past decade, and presents our accumulative experience to overcome these limitations.

### 1.1 Off‐Target: The Main Challenge In CRISPR/Cas9 Specificity

Ensuring the specificity of CRISPR systems remains a significant challenge in genome editing. The primary concern arises from well‐documented evidences that the CRISPR/Cas9 system can induce unintended DNA alterations, known as off‐target effects. These off‐target effects can result in mutations at nontarget genomic sites, potentially leading to adverse outcomes. Cas9 targeting fidelity is primarily determined by the 20‐nucleotide single‐guide RNA (sgRNA) and the PAM.^[^ ^18^, ^19^ ^]^ Despite this, off‐target cleavage can occur at sequences with up to 3–5 bp mismatches in the PAM‐distal region.^[^ ^20^ ^]^ These off‐target events arise from the genetic architecture, the inherent biochemical properties of the CRISPR/Cas9 machinery, experimental design choices, and the biological context of the target cells.^[^ ^21^ ^]^ Therefore, a thorough understanding of the mechanism underlying off‐target phenomenon is crucial for developing effective strategies to minimize them.

### 1.2 What Factors Mediate CRISPR/Cas9‐Induced Off‐Target Effects?

Understanding the key determinants of off‐target effects is crucial for optimizing CRISPR technology and enhancing its specificity. The primary factor influencing off‐target effects in CRISPR/Cas9 editing is mismatch tolerance in the pairing between the guide RNA (gRNA) and target DNA. This tolerance enables Cas9 to bind and cleave DNA sequences that do not perfectly match the gRNA, leading to unintended edits. The likelihood of mispairing is influenced by a combination of biochemical, genetic, and cellular factors, which collectively shape the specificity of CRISPR/Cas9 editing.

#### 1.2.1 Genetic Variations (GVs)

**Figure 1:**
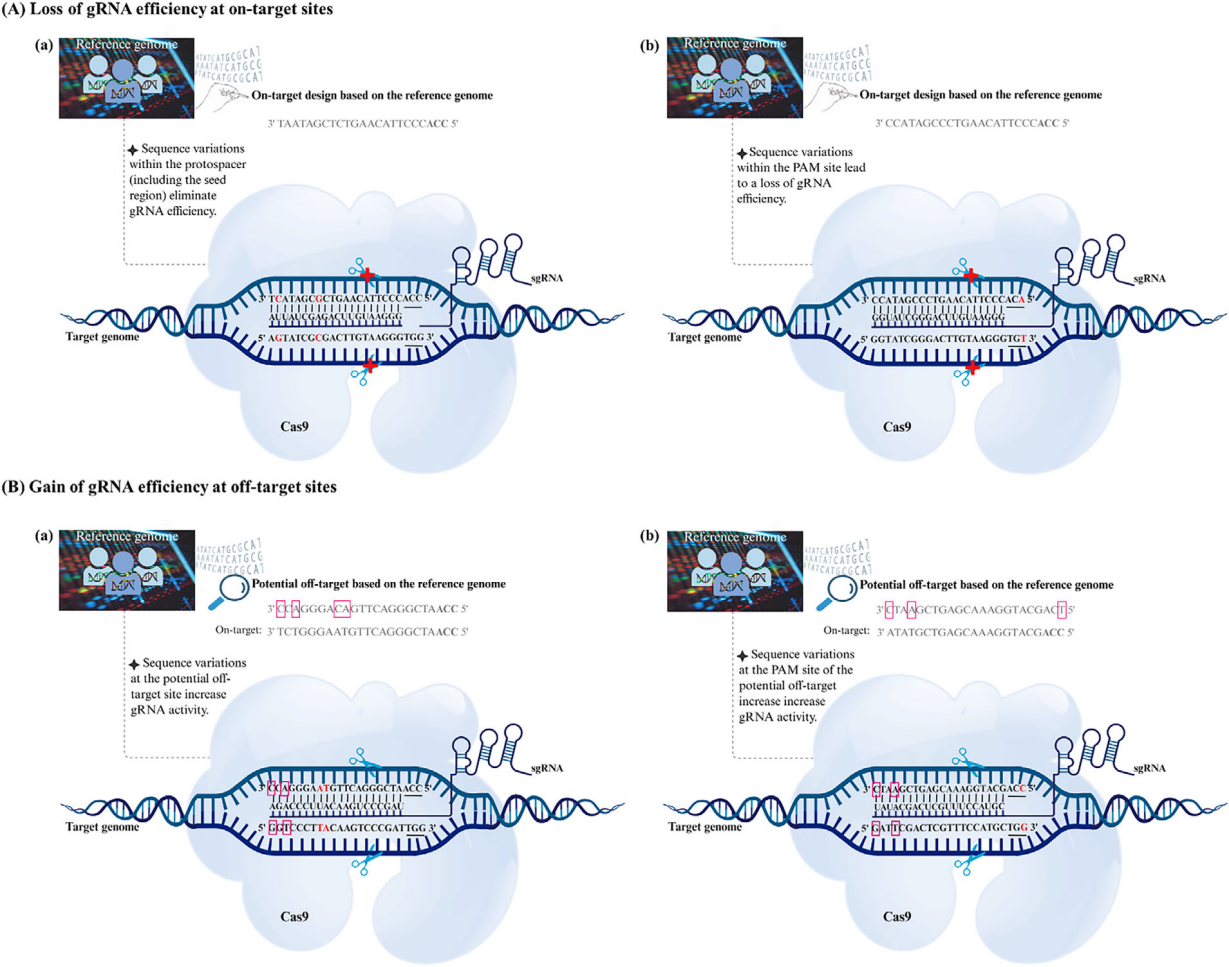
Impact of genetic variation (GV) on CRISPR‐based targeting. A) GVs cause loss of gRNA efficiency at on‐target sites. a) GVs within the protospacer (near the seed region) result in loss of gRNA activity, failure in gRNA recognition and pairing that impair nuclease binding and DNA cleavage. b) GVs in the PAM site lead to a complete loss of gRNA activity and failure in DNA cleavage. B) GVs cause gain of gRNA efficiency at off‐target sites. a) GVs at potential off‐target sites can create novel off‐targets and PAM sites, resulting in increasing the potency of gRNA recognition and pairing, as well as nuclease binding and DNA cleavage. b) GVs at the PAM sequence of potential off‐target sites can create novel off‐targets, resulting in increased potency of gRNA recognition and pairing, as well as nuclease binding and DNA cleavage. GVs are highlighted in red. Bases in colored squares indicate mismatches between the on‐target and off‐target, which are predicted based on the reference genome. Vertical lines present the base pairing between the gRNA and the corresponding matching sequence at the on‐target.

GVs in the target DNA significantly contribute to mismatch tolerance in CRISPR/Cas9 editing. These variations can arise from single nucleotide polymorphisms (SNPs), insertions, deletions, or structural variations. When the gRNA is designed based on a reference genome, any existing GVs in the actual target site can mislead the selection of perfectly matched sgRNAs, introducing mismatches between the designed sgRNA and the intended target site.^[^ ^22^, ^23^, ^24^ ^]^ This mispairing can decrease binding affinity, thereby reducing the specificity of the Cas9 nuclease (**Figure** **1**A). Additionally, GVs within the PAM region can destroy the PAM site at the on‐target loci, rendering the target inaccessible or incompatible with the selected CRISPR nuclease. Conversely, GVs can also introduce novel PAM sites at unintended loci, expanding the targeting range for CRISPR‐based genome editing (Figure 1B). While this expansion can be advantageous, it also carries the risk of generating new off‐target sites.^[^ ^25^ ^]^ Moreover, screening methods such as targeted amplicon sequencing may overlook variant‐induced off‐target mutations and on‐target errors.^[^ ^26^, ^27^ ^]^ If off‐targets are not adequately considered, this can lead to biased assessments of editing efficiency and specificity.^[^ ^28^ ^]^ Such biases pose a significant challenge in distinguishing between genuine editing events and spontaneous mutations, complicating the interpretation of CRISPR/Cas9 efficiency.

#### 1.2.2 Relaxed PAM Requirements

The PAM is essential for Cas9 activity, serving as a binding signal for DNA cleavage. In the case of SpCas9, the canonical PAM is NGG. Cas9 exhibits relaxed PAM requirements, tolerating suboptimal PAMs like NAG or NGA.^[^ ^29^ ^]^ This flexibility has twofold implications: on the one hand, it expands the CRISPR system's utility by enabling the targeting of sequences with noncanonical PAMs. On the other hand, it amplifies the risk of unintended edits at loci with suboptimal PAMs and protospacer similarities. Even with the existence of a valid PAM, mismatches between the gRNA and the protospacer can allow off‐target binding and cleavage.^[^ ^30^ ^]^ Notably, mismatches distal to the PAM (e.g., in regions farther from the NGG motif) are generally more tolerable than those near the PAM, which typically disrupt editing activity. Although mismatches occurring in the seed region (positions 1–12 adjacent to the PAM) typically diminish enzymatic activity. Specific conditions, such as prolonged exposure to the gRNA or elevated enzyme concentrations, may still permit cleavage at these suboptimal sites.^[^ ^31^ ^]^ In addition, partial complementarity, combined with a permissive PAM, can thus lead to off‐target effects.^[^ ^32^ ^]^

#### 1.2.3 Enzymatic Behavior of Cas9

The enzymatic behavior of Cas9 that drives CRISPR/Cas9‐induced off‐target effects is rooted in its biochemical flexibility and interaction dynamics with DNA. While CRISPR/Cas9 technology is revolutionary for genome editing, its specificity is inherently influenced by Cas9's ability to tolerate mismatches.^[^ ^29^, ^33^ ^]^ This mismatch tolerance is impaired by structural variances such as RNA or DNA bulges, which facilitate Cas9's engagement with sequences that deviate from the intended target.^[^ ^34^ ^]^ Moreover, environmental conditions, including temperature and buffer composition, significantly influence Cas9 binding affinity and cleavage efficiency.^[^ ^35^ ^]^ A comprehensive understanding of these dynamics is essential for optimizing CRISPR‐mediated editing while minimizing off‐target effects, ultimately enhancing the specificity and safety of this powerful genomic tool.

### 1.3 How to Minimize Off‐target Effects?

To balance high editing efficiency with minimal off‐target effects in CRISPR/Cas9 applications, a multifaceted approach addressing GVs, relaxed PAM specificity, and Cas9's enzymatic behavior is essential. Below are integrated strategies to optimize specificity and safety.

#### 1.3.1 Pre‐Editing Considerations

In the chase of successful CRISPR/Cas9 applications, careful pre‐editing considerations are important to enhance editing accuracy and minimize off‐target effects. These preliminary steps, focusing on comprehensive genomic understanding and the strategic use of computational tools, lay the basis for precise and safe genome editing. A critical initial step for accurate CRISPR/Cas9 editing involves a thorough genomic analysis of the target genome. This analysis includes identifying and characterizing GVs such as SNPs, indels, and copy number variants to enable precise targeting and unbiased evaluation of editing outcomes.^[^ ^36^ ^]^ The method of genomic analysis depends on the application. The CRISPR‐based therapies in human, where safety and precision are paramount, direct sequencing of the patient's genome is critical.^[^ ^23^, ^37^ ^]^ However, in other organisms, such as plants, sequencing every individual is often not feasible due to scale and cost. Instead, using highly conserved inbred lines with known sequences helps minimize errors from GVs.^[^ ^38^ ^]^ Furthermore, a meticulous evaluation of GVs that impacts PAM sites is vital for assessing potential risks and designing effective therapeutic strategies. Employing advanced computational tools is crucial for enhancing editing specificity. Algorithms such as CRISPOR,^[^ ^39^ ^]^ CHOPCHOP,^[^ ^40^ ^]^ and CRISPRoff^[^ ^41^ ^]^ can predict off‐target sites and rank sgRNAs based on their specificity. It is advisable to avoid protospacers that have predicted off‐targets near critical genes to minimize unintended effects. **Table** **1** summarizes various widely used tools for off‐target prediction. These tools assist in identifying and mitigating the risk of unintended genome modifications. By integrating comprehensive genomic analyses and leveraging computational tools, researchers can significantly optimize pre‐editing strategies, ultimately improving the efficacy and safety of CRISPR/Cas9 applications.

**Table 1:** Widely used tools for off‐target prediction in CRISPR editing.

Tool Name	Key features	URL
**CRISPRoff**	Off‐target prediction, mismatch scoring, genome reference input.^[^ ^41^ ^]^	https://rth.dk/resources/crispr
**CRISPOR**	On/Off‐target scoring, PAM consideration, cleavage efficiency prediction.^[^ ^39^ ^]^	http://crispor.tefor.net/
**Cas‐OFFinder**	Fast genome‐wide search, supports various Cas9 orthologs, large genome analysis, detailed mismatch information.^[^ ^272^ ^]^	http://www.rgenome.net/cas‐offinder/
**CCTop**	Easy‐to‐use tool for mismatch‐based scoring and customizable PAM prediction.^[^ ^273^ ^]^	https://cctop.cos.uni‐heidelberg.de:8043/
**CRISPR‐P**	Web‐based tool for predicting sgRNA off‐target sites and their locations specifically in plant genomes.^[^ ^274^ ^]^	http://cbi.hzau.edu.cn/cgi‐bin/CRISPR
**CHOPCHOP**	A versatile tool for designing sgRNAs for various CRISPR systems, off‐target predictions, on‐target activity scoring.^[^ ^40^ ^]^	https://chopchop.cbu.uib.no/
**CRISPRseek**	R/Bioconductor package, mismatch tolerance, alternative PAMs.^[^ ^275^ ^]^	https://bioconductor.org/packages/release/bioc/html/CRISPRseek.html
**DeepCRISPR**	AI‐driven off‐target prediction, deep learning‐based analysis, visual outputs.^[^ ^276^ ^]^	http://www.deepcrispr.net/.
**OffScan**	Machine learning‐based CRISPR/Cas9 off‐target prediction.^[^ ^277^ ^]^	
**COSMID**	Optimized gRNA design, minimizes off‐target effects.^[^ ^278^ ^]^	http://crispr.bme.gatech.edu
**CROP**	Plant‐specific, validated sgRNA database.^[^ ^279^ ^]^	https://github.com/vaprilyanto/crop
**CRISPRme**	Considers SNVs, indels, haplotypes, PAM mismatches.^[^ ^280^ ^]^	http://crisprme.di.univr.it/

#### 1.3.2 Optimizing sgRNA Design

Effective sgRNA design is pivotal for reducing CRISPR/Cas9 off‐target effects, requiring a balance between hybridization stability and sequence specificity. One key strategy is to prioritize sgRNAs in low‐variation genomic regions and avoid sequences near SNPs or copy‐number variants, as these can increase the risk of off‐target binding, particularly in genetically diverse populations.^[^ ^42^, ^43^ ^]^ Utilizing tools like SNP‐CRISPR^[^ ^44^ ^]^ can help identify sgRNAs that are at higher risk for off‐target interactions. Additionally, maintaining a balanced GC content between 40% and 60% enhances hybridization stability and reduces nonspecific binding,^[^ ^45^ ^]^ further improving the specificity of the editing process. Avoiding shorter or truncated sgRNAs is also important, as short sgRNAs often exhibit reduced specificity due to decreased binding stability.^[^ ^46^ ^]^ By implementing these strategies in sgRNA design, researchers can significantly enhance the specificity and efficacy of CRISPR/Cas9 gene editing, thereby minimizing off‐target effects.

#### 1.3.3 Utilizing Engineered Cas9 Variants

The wild‐type SpCas9 nuclease has been broadly adapted for genome editing due to its unprecedented DNA cleavage capability, though it is associated with significant off‐target effects.^[^ ^47^ ^]^ To address this limitation, various SpCas9 variants with enhanced specificity have been developed, including enhanced SpCas9,^[^ ^30^, ^48^ ^]^ hyper‐accurate Cas9,^[^ ^49^ ^]^ and high‐fidelity SpCas9.^[^ ^50^ ^]^ These engineered variants significantly reduce off‐target effects and maintain editing efficiency. Other high‐fidelity but lower‐activity variants, such as HeFSpCas9, which has improved specificity—also provides high activity in editing applications.^[^ ^51^ ^]^ Delivery of high‐fidelity variants, like HiFi Cas9, have shown improved specificity when delivered as ribonucleoprotein (RNP) complexes, making them particularly beneficial for therapeutic applications.^[^ ^52^ ^]^ Another innovative approach involves using Cas9 nickases (nCas9), which cut only one DNA strand. When paired with dual sgRNAs, nCas9 systems can create single‐strand breaks that greatly reduce off‐target effects, achieving a more than 100‐fold reduction in double‐strand breaks (DSBs).^[^ ^53^ ^]^ While using paired nCas9 to create double‐strand breaks can increase Cas9 specificity.^[^ ^53^, ^54^ ^]^ Moreover, substituting SpCas9 with alternative nucleases, such as Cpf1 (Cas12a), can further reduce off‐target risks. Cpf1 recognizes T‐rich PAMs and exhibits staggered DNA cleavage, making it a preferable choice for applications that demand higher specificity.^[^ ^55^ ^]^ Despite the advancements in engineered Cas9 proteins and alternative nucleases, the complete elimination off‐target activities remain a significant challenge in genome editing.

### 1.4 How Does a Restricted Repair‐Specific Decision‐Making Pathway Influence the Precision of CRISPR Editing?

The leading breakthrough of CRISPR technology is the ability of Cas nucleases to introduce DSBs at specific genomic loci, activating cellular repair pathways. The choice between these pathways, primarily nonhomologous end joining (NHEJ) and homology‐directed repair (HDR), is crucial for the precision of genetic modifications,^[^ ^56^ ^]^ in addition to the microhomology‐mediated end joining (MMEJ), single‐strand annealing (SSA), and homology‐mediated end joining (HMEJ) pathways. At the single‐cell level, at least one repair mechanism typically responds to DSBs, often favoring NHEJ. This process directly ligates DNA ends through error‐prone repair, leading to random deletions or insertions at the break site, which usually results in loss‐of‐function mutations.^[^ ^57^ ^]^ While this error‐based strategy has aided the application of CRISPR‐induced gene knockout experiments on a wide scale, it does not enable accurate, error‐free genetic engineering. In contrast, HDR can precisely introduce desired sequences at specific loci during the DNA synthesis phase (S) and the preparatory phase for mitosis (G2). Additionally, the MMEJ and SSA pathways can also mediate DSB repair with varying degrees of precision, allowing for targeted integrations using specially designed donor templates. Similarly, HMEJ provides a flexible alternative for gene modifications when longer homologous sequences are not feasible. These capabilities allow for various sophisticated applications, including gene correction, gene replacement, point mutations, and gene knock‐ins.^[^ ^58^ ^]^ However, the weak endogenous homology‐dependent repair mechanisms and the requirement to deliver donor templates have limited their application.

The mechanism by which cells select an appropriate repair pathway is a fundamental aspect of determining repair/editing outcomes. The decision‐making process for the corresponding repair pathway relies on several factors, including the nature of the DNA ends and the specific phase of the cell cycle. These factors influence repair‐specific decision‐making pathway by either inhibiting or promoting resection, thereby affecting the choice of the repair pathway or by the concurrency of the cell cycle phase. By highlighting these biological contexts, our understanding of how cells operate repair‐specific decision‐making pathway will be expanded, shedding light on the relationship between factors involved in DNA end resection and the cell cycle phase, ultimately enhancing editing precision.

#### 1.4.1 Effect of End Resection on the Precision of CRISPR Editing

**Figure 2:**
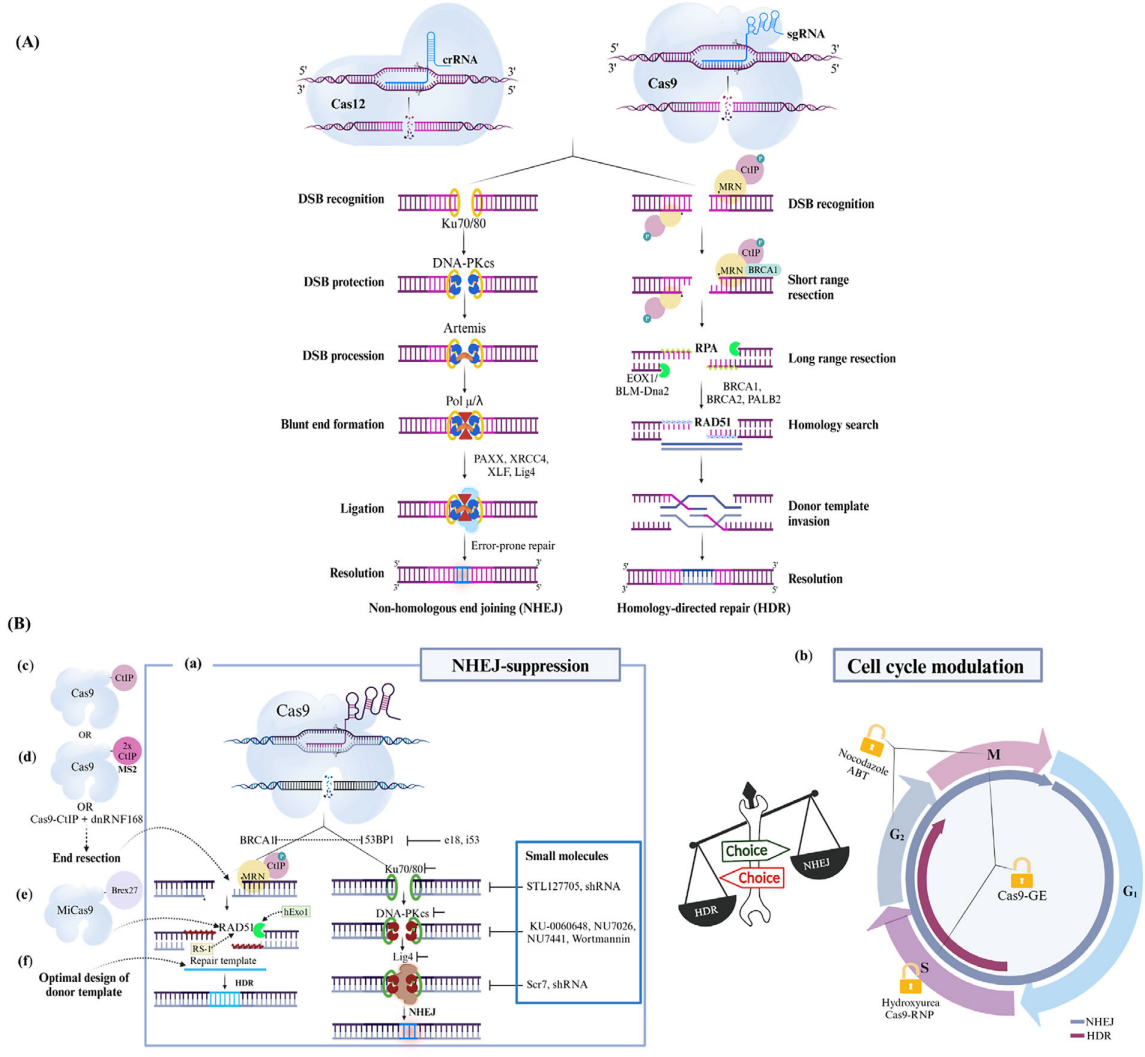
Repair‐specific decision‐making choice toward HDR. A) A schematic illustration of DNA repair pathways. The Cas9 or Cas12 nucleases, guided by a specific sgRNA, cause a DSB at a specific site within the genome. The NHEJ repair pathway is initiated when Ku70/80 proteins recognize and bind to the break ends. Following end recognition, end protection and processing begin when Ku recruits DNA‐dependent protein kinase catalytic subunit (DNA‐PKcs), which has a tight affinity to the DNA ends, forming a stable complex that protects the DNA ends from resection and further damage. This recruitment enhances the affinity of the subsequent enzymatic components, forming a highly stable complex that phosphorylates downstream NHEJ proteins, including artemis, XRCC4, and XLF. The latter nucleases process the incompatible and chemically modified ends, making them ligatable and facilitating end ligation by the DNA ligase IV complex (Lig4). These reactions promote the alignment of DNA ends in an error‐prone pattern, resulting in small indels that can generate a loss‐of‐function mutation. This forms the basis of NHEJ‐based error‐prone editing. The HDR pathway involves sophisticated repair processing mechanisms led by 5′‐3′ DNA end resection to form 3′ single‐stranded DNA overhangs and the presence of undamaged sister chromatid or donor DNA. End resection begins when the MRN (MRE11‐RAD50‐NBS1) complex recruits CtBP‐interacting protein (CtIP), leading to the generation of short single‐stranded tails. Exo1 and the DNA2/BLM complex then perform long‐range resection, resulting in 3′ ssDNA tails. RPA stabilizes the ssDNA, which RAD51 subsequently replaces with the assistance of recombination mediators such as BRCA1, BRCA2, and PALB2. RAD51 forms nucleoprotein filaments on the ssDNA, facilitating homology search and strand invasion of a homologous DNA template. Finally, resolvases process the resolution junction, completing the repair and restoring the chromosome to its original state. B) Strategies to redirect cell's repair‐specific decision‐making choice toward HDR. The process of pathway decision‐making between NHEJ and HDR is influenced by several repair factors. As NHEJ is the default repair pathway, researchers aim to manipulate the factors that govern the transition between NHEJ and HDR to selectively enhance HDR over NHEJ. a) Exploiting NHEJ inhibition is a main strategy that favors the HDR pathway. This includes targeting 53BP1, a key promoter of the NHEJ pathway, through methods such as using ubiquitin variants (i53) or expressing factors that displace 53BP1 like (e18). It also involves using small molecule inhibitors against Ku70/80, DNA‐PKcs and LIG4, which are critical components of the NHEJ machinery. b) Manipulating the cell cycle to favor HDR that is active during the S/G2/M phases, in contrast to NHEJ, which operates throughout the cell cycle. This strategy involves the use of compounds to block cells in HDR‐permissive phases. c) Developing a Cas9‐CtIP fusion protein, d) dual molecules of CtIP (2x CtIP) to Cas9 using MS2 tagging, e) or fusing Cas9 with a small motif of BRCA2 (Brex27), forming MiCas9, enables end‐resection. f) Rational design of the ssODN donor template to be complementary to the non‐target strand can also improve the efficiency of precise editing.

End resection plays a crucial role in determining the choice of DNA repair pathways, significantly influencing the precision of CRISPR/Cas9 editing. Understanding the factors that regulate end resection and repair pathway choice is essential for enhancing CRISPR editing precision. During the repair of DSBs, two types of protein regulators govern the balance between NHEJ and HDR: anti‐resection factors and resection initiators. Anti‐resection proteins, such as 53BP1, inhibit end resection and promote NHEJ by binding to DSB ends. When these factors are active, limited or no resection occurs, favoring NHEJ.^[^ ^59^ ^]^ In contrast, resection initiators, including the M‐R‐N complex and BRCA1, facilitate end resection, which is essential for activating HDR.^[^ ^60^ ^]^ Extensive end resection generates overhangs that aid in the search for homologous sequences, such as sister chromatids or exogenous DNA templates, thereby guiding accurate repair. By manipulating the activity of these protein regulators, scientists aim to shift the balance toward HDR, improving the accuracy and efficiency of CRISPR/Cas9‐based genome editing. This strategic control of end resection holds great potential for precise genetic modifications in various domains, including fundamental research, biotechnology, and therapeutic applications. A detailed explanation of the mechanisms of NHEJ and HDR repair pathways is illustrated in **Figure** **2**A.

#### 1.4.2 Cell Cycle Dynamics Shape the Precision of CRISPR/Cas9 Editing

The cell cycle phase at which DSBs occur is a critical determinant of the choice between DNA repair pathways. As cells progress through the cycle, the preference for either NHEJ or HDR shifts.^[^ ^61^, ^62^ ^]^ During the S and G2 phases, when sister chromatids are available, HDR is favored due to the presence of repair templates. These phases allow for precise editing, as the resected DNA ends can engage in homology search and strand invasion, leading to accurate repair using the sister chromatid. In contrast, NHEJ is active throughout the cell cycle, competing with HDR even during these critical phases. The rapid response of the NHEJ pathway, primarily due to the quick binding of the Ku complex, often results in immediate repair,^[^ ^63^ ^]^ which can lead to error‐prone outcomes and loss‐of‐function mutations.

Understanding how cells regulate repair pathways during different cell cycle phases is essential for enhancing the precision of CRISPR/Cas9 editing. For instance, during the G1 phase, proteins like 53BP1 and BRCA1 play pivotal roles in determining pathway choice. Phosphorylated 53BP1 binds to DSB ends, inhibiting end resection and favoring NHEJ, while BRCA1 facilitates the recruitment of HDR machinery.^[^ ^64^ ^]^ As cells transition into the S and G2 phases, BRCA1 antagonizes 53BP1, promoting end resection and thereby enhancing the efficacy of HDR.^[^ ^65^ ^]^ This shift is crucial for initiating HDR, as the resected DNA ends can then engage in homology search and strand invasion, utilizing the sister chromatid as a template. Understanding the coordination between the cell cycle and repair pathway choice is essential for precise repair.

### 1.5 How Can Path Redirection Strategies Drive Repair Pathway Choice Toward Enhanced Precision?

The repair‐specific decision‐making process is regulated by several factors that govern the transition between NHEJ and HDR. Researchers aim to manipulate these factors to enhance HDR over NHEJ, striving for error‐free repair, particularly in the fields of gene therapy and genome editing, where precision is paramount. In the following section, we will highlight some of these strategies.

#### 1.5.1 Boosting CRISPR/Cas9 HDR Precision: A Strategy of Suppressing Key NHEJ Factors

The balance between NHEJ and HDR is regulated as cells transition from G1 to S/G2 phases. High NHEJ activity can inhibit HDR progression; thus, suppressing NHEJ allows for compensatory homology‐directed repair of DSBs.^[^ ^66^, ^67^ ^]^ Researchers have successfully channeled repair choices toward HDR by inhibiting key NHEJ factors. For example, knocking out Lig4 enhances homologous recombination,^[^ ^68^ ^]^ and suppressing Ku70/80 or DNA‐PKcs also boosts HDR efficiency.^[^ ^69^, ^70^ ^]^ Repair factors from both pathways are recruited independently; however, proteins like ATM and DNA‐PKcs participate in both.^[^ ^71^ ^]^ This insight helps scientists understand the trade‐off between NHEJ and HDR.

Protein inhibitors, like small molecules, have shown the potential to enhance HDR over NHEJ, thereby enhancing CRISPR/Cas9 precision. Inhibiting DNA‐PK kinase activity, for instance, by chemical inhibitors increases HDR.^[^ ^66^ ^]^ The Lig4 inhibitor Scr7 effectively enhances CRISPR/Cas‐mediated HDR in diverse organisms.^[^ ^72^ ^]^ The small molecule i53 also reduces NHEJ activity by preventing 53BP1 binding, further enhancing HDR.^[^ ^73^ ^]^ Additional small molecule inhibitors targeting DNA‐PKcs have also been documented, including wortmannin,^[^ ^74^ ^]^ NU7441,^[^ ^75^ ^]^ KU‐0060648,^[^ ^76^ ^]^ NU7026,^[^ ^70^, ^77^ ^]^ M3814,^[^ ^78^ ^]^ VX‐984,^[^ ^79^ ^]^ and AZD7648.^[^ ^80^ ^]^ Notably, AZD7648 has demonstrated remarkable enhancements in HDR efficiency. These inhibitors provide valuable tools for enhancing HDR, ultimately improving the precision and efficiency of CRISPR/Cas‐mediated gene editing across multiple applications. Figure 2Ba illustrates strategies for enhancing CRISPR/Cas9 precision by suppressing key NHEJ factors.

#### 1.5.2 Enhancing CRISPR Precision through Cell Cycle Synchronization for Improved HDR

Synchronizing the cell cycle is crucial for precise repair, especially in the S/G2 phases where HDR is most active. Researchers often use chemical agents to selectively arrest cells at specific phases, where sister chromatids are available as repair templates. This timely intervention helps bias the repair pathway toward HDR, thus enhancing repair precision. For example, Lin et al. combined nucleofection of Cas9 RNP with chemical inhibitors, improving HDR efficiency by three to six times.^[^ ^81^ ^]^ Synchronized Cas9 expression in S/G2/M phases, while suppressed in G1, increased HDR rates by up to 87% through the Cas9‐Geminin fusion.^[^ ^82^ ^]^ Researchers also utilized early embryo‐specific promoters to enhance HDR efficiency in gene editing.^[^ ^83^, ^84^ ^]^ Inhibitors like hydroxyurea can arrest cells in the S phase (Figure 2Bb), promoting CRISPR/Cas9‐mediated HDR across various living cells.^[^ ^85^, ^86^, ^87^ ^]^ However, these methods may have limitations for in vivo applications due to potential toxicity. Cell cycle synchronization can also be achieved by regulating key genes, such as CtIP (Figure 2Bc), which promotes DNA end resection, thus activating the HDR pathway even in the G1 phase.^[^ ^88^, ^89^, ^90^ ^]^ This modulation can facilitate precise genome editing and repair processes.

#### 1.5.3 Enhancing CRISPR Precision: Fine‐tuning Editing Factors to Favor HDR Repair

Various strategies have been employed to fine‐tune cell repair components and create a favorable cellular environment to channel repair choice for CRISPR‐mediated HDR. These strategies enhance control over repair pathway preference, enabling precise and efficient genome editing. Below are key methods to promote HDR:

##### 1.5.3.1 Spatial and Temporal Co‐localization of HDR‐Promoting Factors

Fusing and co‐delivering Cas9 with HDR‐enhancing proteins can significantly improve HDR efficiency by stimulating HDR effectors to localize at the site of DSBs. One example is the Cas9‐RS‐1 fusion, which boosts RAD51 activity and increases knock‐in efficiency by 2–5 times.^[^ ^91^ ^]^ Other enhancers, like e18 and DN1S, prevent NHEJ‐promoting factors from localizing at DSB sites, enhancing HDR assembly.^[^ ^92^, ^93^ ^]^ Introducing RAD51 alongside Cas9 enhances homologous recombination while reducing NHEJ.^[^ ^94^ ^]^ HDR enhancement can also be achieved by promoting 5′‐3′ end resection via fusing Cas9 with truncated Exo1 or a minimal N‐terminal fragment of CtIP (Figure 2Bc), increasing knock‐in rates.^[^ ^95^, ^96^ ^]^ Additionally, combining Cas9‐CtIP with a dominant negative mutant of RNF168 reduces indels and improves HDR.^[^ ^97^ ^]^ In line with this, recruiting dual molecules of CtIP to Cas9 using MS2 tagging elevated the HDR to indel ratio^[^ ^98^ ^]^ (Figure 2Bd). However, co‐expressing certain fusions may lead to redundancy,^[^ ^99^ ^]^ highlighting the need for careful selection of manipulated elements. Moreover, MiCas9, fused with a BRCA2 motif, effectively attracts Rad51 and achieves peak HDR rates^[^ ^100^ ^]^ (Figure 2Be).

##### 1.5.3.2 Optimizing Donor Template Design

Single‐stranded oligonucleotides (SSOs) serve as HDR repair templates. Optimizing SSO design, considering length and sequence, can enhance HDR efficiency (Figure 2Bf). Increasing donor availability at DSBs is crucial; one method involves fusing SaCas9 to SNAP‐tag technology, improving donor proximity and HDR rates.^[^ ^101^, ^102^ ^]^ Similarly, fusing Cas9 with avidin by biotinylated SSOs and a flexible amino acid joiner enhances donor accumulation at break sites.^[^ ^103^ ^]^ However, engineering donor sequences can be complex and costly. Approaches like linking SSOs via an endonuclease recognition sequence simplify this process.^[^ ^104^ ^]^ Additionally, engineered Cas9 variants, such as those attached to VirD2 relaxase, can significantly enhance gene knock‐in precision, especially in plants.^[^ ^105^ ^]^ While donor enrichment improves specificity, it may also lead to repetitive insertions and off‐target effects, necessitating further investigation.

##### 1.5.3.3 Harnessing Epigenetic Modulation

Epigenetic modulation involves manipulating chromatin structure and histone modifications to regulate gene expression. Chromatin exists in loosely packed (euchromatin) or tightly packed (heterochromatin) forms, regulated by linker histones and epigenetic modifiers like methylation and acetylation. This packaging controls the accessibility of transcriptional machinery. However, the influence of these chromatin states on HDR is not fully understood. Pioneer transcription factors and chromatin remodeling complexes play a key role in facilitating epigenetic editing. For example, fusing Cas9 with histone epigenetic modifiers, such as PRDM9, leverages histone modifications to promote HDR over NHEJ at DSBs.^[^ ^106^ ^]^ In line with this strategy, known epigenetic regulators identified to enhance HDR are potential candidates for integration with the CRISPR system to improve the precision of CRISPR‐mediated editing. These include long noncoding RNAs,^[^ ^107^ ^]^ histone deacetylases,^[^ ^108^ ^]^ and SWI/SNF^[^ ^109^, ^110^ ^]^ complexes. Understanding the interplay between chromatin dynamics, DNA repair pathways, and gene editing is essential for the development of precise editing tools.

### 1.6 The Delivery Dilemma of CRISPR/Cas9 Components in Mediating Efficient and Accessible Editing

The successful application of the CRISPR/Cas system for gene editing depends on the efficient delivery of its components: the Cas nuclease and gRNA. However, delivering these elements to the target site within the nucleus is a complex task, especially for cell types like plant cells with tightly regulated membrane permeability. The large size of CRISPR/Cas components complicates their packaging into viral vectors, such as adeno‐associated viruses (AAVs), and limits their ability to cross cell membranes and reach target DNA.^[^ ^111^ ^]^ Additionally, maintaining the stability and persistence of the CRISPR/Cas components over time is another concern for effective gene editing.

The potential risks of CRISPR/Cas delivery systems pose significant safety concerns across humans, animals, and plants. Despite its promise, issues like immune responses from viral vectors can lead to inflammation or autoimmune disorders.^[^ ^112^ ^]^ Some chemical carriers may also be genotoxic,^[^ ^113^ ^]^ increasing cancer risk. While the long‐term effects of CRISPR/Cas9 gene editing are under investigation, ethical dilemmas, especially regarding germline editing, could influence future generations. Similar risks, including off‐target effects and immune responses, are present in animals and plants, alongside environmental worries about modified organisms. Regulatory challenges and consumer acceptance remain crucial in agricultural contexts. Researchers actively strive to mitigate these risks with enhanced targeting and safer delivery methods.

### 1.7 What are Key Factors for Efficient CRISPR/Cas Delivery?

The delivery of CRISPR components involves two main factors: i) the format of CRISPR/Cas and ii) the delivery vehicle. The optimal delivery vehicle is largely determined by the specific application and the chosen CRISPR/Cas format, necessitating a tailored toolkit of techniques for effective delivery.

#### 1.7.1 Optimizing CRISPR/Cas Delivery: Choosing the Right Format for the Mission

CRISPR components can be delivered in three main formats: i) plasmid DNA, ii) mRNA, or iii) a ribonucleoprotein (RNP) complex. The delivery format influences the expression of the Cas nuclease and its exposure duration, affecting off‐target activity and editing efficiency. Plasmid DNA, a persistent format, typically results in higher off‐target activity compared to transient formats like mRNA and RNP, which show a quick peak in expression followed by a decline, minimizing prolonged off‐target effects and genotoxicity.^[^ ^31^ ^]^ RNP delivery has proven highly efficient in editing target sequences, while mRNA and DNA plasmids can trigger innate immune responses, posing safety risks. Thus, regulating the expression of CRISPR/Cas components is crucial for enhancing nuclease activity. Strategies such as small molecules,^[^ ^114^, ^115^ ^]^ split‐Cas9,^[^ ^116^ ^]^ and magnetic nanoparticles^[^ ^117^ ^]^ have been employed to address these issues. Recently, CRISPR RNPs were successfully delivered to mouse lung epithelial cells using shuttle peptides, achieving rapid and prolonged editing, along with a swift peptide decline.^[^ ^118^ ^]^

#### 1.7.2 Strategic Selection of CRISPR/Cas Delivery Vehicles

“Delivery vehicles” refer to systems that transport the CRISPR/Cas complex into the target cells. The CRISPR/Cas delivery system involves three approaches: viral vectors, physical methods, and chemical methods. Viral vectors, like AAV,^[^ ^119^ ^]^ adenovirus,^[^ ^120^ ^]^ lentivirus,^[^ ^121^ ^]^ and baculovirus‐based vectors,^[^ ^122^ ^]^ are effective for long‐term editing,^[^ ^123^ ^]^ but they pose risks of off‐target effects, raising specificity and safety concerns. Additionally, they have inherent shortcomings, including insertional mutagenesis, packaging size constraints, and immunogenicity.^[^ ^124^ ^]^ Nonviral vectors have gained traction, employing organic or inorganic materials. While physical methods, such as microinjection and electroporation, disrupt the cell membrane to facilitate CRISPR delivery, this may damage cells, posing safety concerns. To overcome the limitations and drawbacks of physical and viral methods, Chemical methods offer lower immunogenicity and greater scalability, though they face challenges in stability and precise delivery. A detailed overview of these delivery approaches is provided in **Table** **2**.

**Table 2:** Summary of CRISPR delivery systems and their applications.

Type of Delivery System		Cargo Format	Description	Cells/Tissues	Advantages	Limitations
**Viral Vectors**	Adeno‐associated virus (AAV)	DNA	Utilizes AAV to deliver CRISPR components.^[^ ^281^ ^]^	Somatic cells	High efficiency, low immunogenicity, broad tissue tropism.	Limited cargo size (∼4.7 kb), potential immune response with repeated dosing.
	Lentivirus	RNA	Uses lentiviruses to integrate CRISPR components.^[^ ^282^ ^]^	Somatic & germline cells	High efficiency, stable integration, can infect dividing and non‐dividing cells.	Risk of insertional mutagenesis, potential for oncogenesis, immune response.
	Adenovirus	DNA	Utilizes adenoviruses to deliver CRISPR components, often episomally without integration.^[^ ^283^ ^]^	Somatic cells	High transduction efficiency, large cargo capacity (up to ∼8 kb).	High immunogenicity, transient expression not suitable for long‐term applications.
	Baculovirus	DNA/ proteins	Uses baculoviruses to deliver CRISPR components.^[^ ^122^, ^284^ ^]^	Somatic cells	High transduction efficiency in insect cells, large cargo capacity.	Limited to certain cell types, immune response in mammalian cells.
**Lipid Nanoparticles**	Cationic lipid nanoparticles	DNA/RNA	Utilizes positively charged lipids to form complexes with negatively charged nucleic acids.^[^ ^153^ ^]^	Somatic cells	High transfection efficiency, easy to formulate.	Potential toxicity, variable efficiency across cell types.
	Ionizable lipid nanoparticles	DNA/RNA	Uses neutral lipids that become positively charged in acidic environments.^[^ ^285^ ^]^	Somatic cells	Reduced toxicity, efficient endosomal escape, enhancing delivery efficiency.	Production complexity, potential immunogenicity.
	Polymer‐coating Lipid Nanoparticles	DNA/RNA	Cationic lipid nanoparticles coated with polymers, like polyethylene glycol (PEG).^[^ ^286^, ^287^ ^]^	Somatic cells	Increased circulation time and stability, reduced immune clearance.	Potential for PEG‐related immune reactions, reduced cell uptake.
	Solid lipid nanoparticles	DNA/RNA	Comprised of solid lipids that encapsulate nucleic acids.^[^ ^288^ ^]^	Somatic cells	High stability, controlled release	Lower transfection efficiency, formulation complexity
	Hybrid lipid‐polymer nanoparticles	DNA/RNA/Proteins	Combines lipids and polymers.^[^ ^289^ ^]^	Somatic cells	Improved stability, enhance delivery efficiency, tunable properties.	Complex synthesis, potential for polymer‐related toxicity.
	Targeted lipid nanoparticles	DNA/RNA	Lipid nanoparticles conjugated with targeting ligands (e.g., antibodies, peptides).^[^ ^290^, ^291^ ^]^	Specific tissues, tumor cells	Enhance delivery to specific cell types, reduced off‐target effects.	Formulation complexity, potential for off‐target effects in non‐target tissues.

Type of Delivery System			Cargo Format	Description	Cells/Tissues	Advantages	Limitations
	**Liposomes**	Lipoplexes	DNA/NA/Proteins	Hybrid nanoparticles combining lipids with polycations to form complexes with nucleic acids.^[^ ^292^ ^]^	Somatic cells	Enhanced stability, co‐delivery capabilities.	Potential toxicity, complex formulation process.
		Stable nucleic‐acid‐lipid particles (SNALPs)		Liposome nanoparticles containing acidic pH‐sensitive lipids to form complexes with nucleic acids.^[^ ^289^ ^]^			
		Lipopolyplexes		Hybrid nanoparticles combining the cationic lipid bilayer onto anionic/ cationic polyplexes to form complexes with nucleic acids.^[^ ^293^, ^294^ ^]^			
		Membrane/core nanoparticles (MCNPs)		Hybrid inorganic nanoparticles combining a core‐shell‐like structure with lipid bilayer to form complexes with nucleic acids.^[^ ^295^ ^]^			

Type of Delivery System		Cargo Format	Description	Cells/Tissues	Advantages	Limitations
**Gold‐based nanoparticles (AuNPs)**		Cas9 (RNPs), Plasmids	Gold nanoparticles functionalized with various molecules (cationic peptides, polymers, etc.).^[^ ^296^ ^]^	HSPCs cells, Tumor Cells, Neurons and Muscle Cells, Various primary and immortalized cell lines	High Delivery Efficiency, Targeted Delivery, Biocompatibility, Versatility, Reduced Off‐Target Effects.	Complex Synthesis, Cost, Stability Issues, Potential Immunogenicity.
**PEG‐mediated transformation**		DNA/RNA	Utilizes polyethylene glycol to facilitate DNA uptake by protoplasts.^[^ ^297^, ^298^ ^]^	Protoplasts	Effective for protoplasts, allows for direct DNA uptake.	Requires careful regeneration into whole plants, limited to protoplasts.
**Cell‐penetrating peptides (CPPs)**		CPP‐conjugated DNA/RNA/Proteins	Uses peptides capable of penetrating cell membranes to deliver CRISPR components into cells.^[^ ^299^ ^]^	Somatic and germline cells	Non‐viral, low immunogenicity, versatile.	Variable efficiency, potential for degradation by proteases, need for optimization of CPPs for different cell types.
**Electroporation**	In vitro Electroporation	DNA/RNA/Proteins	Uses electric pulses to introduce CRISPR components into cultured cells.^[^ ^300^ ^]^	Cell lines, primary cells	High efficiency, suitable for many cell types.	Can be damaging to cells, requires optimization for different cell types.
	In vivo electroporation		Applies electric pulses directly to tissues within a living organism to deliver CRISPR components.^[^ ^301^ ^]^	Somatic tissues (e.g., muscle, liver, brain)	Allows targeted delivery to specific tissues	Invasive, potential tissue damage, requires precise control of pulse parameters
	*Ex vivo* electroporation		Cells are harvested, treated with CRISPR components via electroporation, and then reintroduced into the organism.^[^ ^302^, ^303^ ^]^	Hematopoietic stem cells, T cells	Allows precise control over conditions, high efficiency	Requires cell harvesting and reintroduction, technically complex.
	Utero electroporation		Uses electroporation to introduce CRISPR components into developing tissues of embryos within the uterus.^[^ ^304^, ^305^ ^]^	Neonatal tissues	High efficiency in neonatal tissues, useful for developmental studies.	Limited to neonatal stage, potential for developmental disturbances.
	Embryo electroporation		Electric pulses are applied to embryos to introduce CRISPR components.^[^ ^306^ ^]^	Embryos	Efficient delivery to early‐stage embryos.	Can be technically challenging, potential for low survival rates in embryos.
	Tissue‐specific electroporation		Targets specific tissues within an organism using localized electric pulses.^[^ ^307^ ^]^	Specific tissues (e.g., brain, muscle, leaves).	Targeted delivery, minimizes systemic effects.	Invasive, requires precise targeting, potential tissue damage.
	Microfluidic electroporation		Uses microfluidic devices to apply electric pulses for introducing CRISPR components.^[^ ^308^ ^]^	Cell lines, primary cells	High precision, can handle small volumes, high efficiency.	Requires specialized equipment, optimization needed for different cell types.
	Nucleofection		An advanced electroporation technique tailored to deliver cargoes and CRISPR/Cas9 components directly into the nuclei of mammalian cells.^[^ ^309^, ^310^ ^]^	Primary cells, stem cells, and those difficult‐to‐transfect cells	High transfection efficiency, Direct nuclear delivery, Suitable for hard‐to‐transfect cells, Rapid and reproducible.	Requires specialized equipment, may cause cell damage or death if not optimized, Limited to small‐scale applications.
**Tissue‐specific microinjection**		DNA/RNA/Proteins	Direct injection of CRISPR components into the pronucleus of a fertilized egg,^[^ ^311^ ^]^ sperm cells (intracytoplasmic sperm injection),^[^ ^312^ ^]^ blastocyst stage of embryos,^[^ ^313^ ^]^ retina,^[^ ^314^ ^]^ muscle tissue,^[^ ^315^ ^]^ pollen tube pathway in plants.^[^ ^316^ ^]^	Zygotes, embryos, Blastocyst, Retinal cells, Muscle cells, Pollen cells	High precision, integration into germline, can create animals with targeted genetic modifications, Direct delivery to target cells, minimal systemic exposure.	Labor‐intensive, technically challenging, low throughput, requires skilled operators, limited to certain plant species, technically challenging.
***Agrobacterium*‐mediated transformation**		DNA	Exploits the natural ability of Agrobacterium to transfer DNA into plant cells.^[^ ^317^, ^318^, ^319^ ^]^	Somatic and germline cells.	Widely used for dicotyledonous plants, stable integration.	Limited to certain plant species, regulatory concerns.
**Particle bombardment (biolistics)**		DNA/RNA	Uses high‐velocity metal particles coated with CRISPR components to penetrate cell walls.^[^ ^320^ ^]^	Somatic and germline cells	Broad applicability, can penetrate tough cell walls.	Can cause significant tissue damage, lower transformation efficiency in some cases.
**Magnetofection**		DNA/RNA	Uses magnetic nanoparticles to concentrate nucleic acids.^[^ ^321^, ^322^ ^]^	Specific tissues	Potential for enhanced efficiency in specific tissues.	Experimental, not widely adopted, requires further optimization.
**Sonoporation**		DNA/RNA/Proteins	Uses ultrasound waves to enhance cell membrane permeability.^[^ ^323^ ^]^	Deep tissues, specific cells	Potential for non‐invasive delivery, can target deep tissues.	Experimental, variable efficiency, potential tissue damage.

### 1.8 What are the Tactics for Efficient Delivery of CRISPR/Cas Components?

#### 1.8.1 Manage Packaging Size

Achieving effective in vivo delivery of CRISPR/Cas components is challenging due to packaging size constraints. Strategies include optimizing components with compact Cas proteins like NmeCas9 (≈3.3 kb)^[^ ^125^, ^126^ ^]^ and SaCas9, (≈3.3 kb)^[^ ^127^ ^]^ or using ultracompact Cas proteins (<2 kb), such as Cas12f, Cas12j, Cas12k, Cas12m,^[^ ^128^ ^]^ and TnpB.^[^ ^129^, ^130^ ^]^ Additionally, the split‐Cas9 system, which utilizes self‐cleaving peptides across two AAV vectors, alleviates size limitations.^[^ ^131^, ^132^ ^]^ Another approach is to employ larger‐capacity delivery methods, like LNPs and polymers, which offer greater cargo capacity. Finally, optimizing vector design through improved packaging or nanoparticle encapsulation can further boost delivery efficiency.

#### 1.8.2 Enhance Cellular Uptake

Cellular uptake of macromolecular CRISPR/Cas cargos presents a significant challenge due to the strict permeability of cellular membranes. Various strategies have been employed to facilitate this transport, including electroporation, which improves the internalization of large macromolecular cargos compared to traditional methods.^[^ ^133^ ^]^ Cell‐penetrating peptides (CPPs), such as TAT^[^ ^134^ ^]^ or R9,^[^ ^135^ ^]^ facilitate the internalization of CRISPR components across different cell types. Additionally, encapsulating CRISPR components in optimized LNP formulations with engineered cationic lipids enhances stability, transfection rates, and reduced toxicity.^[^ ^136^ ^]^ Hybrid systems that combine nanoparticles with CPPs also boost uptake and editing efficiency.^[^ ^137^ ^]^ Moreover, incorporating nuclear localization signals (NLS) into CRISPR components promotes their import into the nucleus.^[^ ^138^ ^]^ Likewise, endosomolytic agents can aid in escaping endosomes, increasing the likelihood of nuclear delivery.^[^ ^139^ ^]^ Combining NLS with CPPs enhances both cellular and nuclear targeting.^[^ ^140^ ^]^

#### 1.8.3 Maintain the Stability of CRISPR/Cas Components

The stability of CRISPR components is essential for effective editing. Optimizing formulations and modifying gRNA backbones can enhance resistance to degradation.^[^ ^141^, ^142^, ^143^ ^]^ Additionally, encapsulating CRISPR components in protective carriers like LNPs, liposomes or hydrogels can prolong persistence in the target cells.^[^ ^123^ ^]^ Moreover, employing controlled release systems, such as microparticles, can extend the presence of these components within target cells.^[^ ^144^ ^]^

#### 1.8.4 Enhance Target Specificity/Minimize Off‐Target Effects

Off‐target effects undermine CRISPR specificity. Engineered AAV variants with improved tissue specificity, such as AAV7/8 for liver targeting, show promise in correcting genetic disorders in preclinical trials.^[^ ^145^ ^]^ Developing viral vectors with enhanced tropism strengthens their affinity for specific cell types and subsequently for the nucleus. This can be achieved through screening new variants or modifying the viral capsid using rational design, directed evolution, and engineering the viral genome's cis‐regulatory components, as reviewed in.^[^ ^146^ ^]^ Furthermore, optimizing the composition, size, and surface properties of nanoparticles,^[^ ^147^ ^]^ along with incorporating targeting ligands^[^ ^148^, ^149^ ^]^ or CPPs,^[^ ^150^ ^]^ improves specific delivery. Hybrid systems that combine viral vectors and LNPs, along with innovative formulations such as hydrogels and microparticles, can leverage the strengths of multiple approaches for targeted, controlled, and sustained CRISPR/Cas delivery.

#### 1.8.5 Mitigate Immunogenicity in CRISPR Delivery

The possibility of triggering immune responses presents another significant challenge to the clinical application of CRISPR technology. Using AAVs augments cytotoxicity and initiates immune responses as it provides prolonged expression of Cas9, posing safety concerns. Using immunologically silent delivery vehicles like lipid nanoparticles can help evade immune detection. Factors such as the size and composition of LNPs may trigger responses.^[^ ^151^ ^]^ Thus, co‐administering immunosuppressants and using biocompatible coatings like polyethylene glycol (PEG) can enhance safety.^[^ ^152^, ^153^ ^]^ Furthermore, optimizing the components themselves, including less immunogenic Cas proteins, can eliminate immune reactions. Developing strategies for selective gene editing activation in specific cell types can also help minimize the risk of immunogenicity.^[^ ^154^ ^]^

### 1.9 The PAM: A Necessary but Restrictive Element

CRISPR/Cas9 technology has revolutionized our ability to manipulate genomic DNA with unprecedented precision and programmability. Successful targeting relies on: i) extensive complementarity between the gRNA and the target sequence, and ii) the presence of a PAM sequence. Although several factors like GC content, off‐target similarity, and secondary structure can influence target selection,^[^ ^155^ ^]^ sgRNA design offers greater flexibility. However, the PAM requirement presents a significant challenge. Different Cas variants exhibit distinct PAM requirements, impacting their targeting versatility and applicability. The most robust and commonly used Type II SpCas9 initially recognizes the canonical 5′‐NGG‐3′ PAM sequence.^[^ ^127^ ^]^ The Cas9 nuclease scans DNA for a 5′‐NGG‐3′ sequence before assessing guide‐target complementarity. Consequently, even a perfectly complementary target sequence lacking a PAM will be ignored. This PAM dependency acts as a gatekeeper for CRISPR/Cas targeting, limiting its versatility. With the rapidly growing list of CRISPR applications, the need for greater flexibility and adaptability has become crucial.

One promising approach to address the targeting range limitations of CRISPR/Cas9 is to engineer Cas9 variants with novel PAM specificities. Engineering SpCas9 has resulted in a Cas9‐NG variant with a more relaxed PAM sequence, 5′‐NG‐3′,^[^ ^156^ ^]^ and broader PAM recognition, such as 5′‐NGAN‐3′ and 5′‐NGCG‐3′ (VQR and VRER Cas9), respectively.^[^ ^157^ ^]^ Researchers have also employed phage‐assisted evolution to engineer SpCas9 variants with both high specificity and expanded PAM recognition. These include xCas9, which recognizes a highly specific 9‐bp PAM sequence (5;‐NNNRRTG‐3′),^[^ ^158^ ^]^ and variants that recognize non‐G 5′‐NRNH‐3′ PAMs (R is G or A, and H is C, T, or A).^[^ ^159^ ^]^ SpCas9 protein was engineered through structure‐guided engineering to highly lessen the PAM requirement, resulting in the formation of a highly enzymatically active variant with a 5′‐NGN‐3′ PAM, termed SpG. Further optimization of SpG then resulted in establishing the SpRY variant, which can edit targets with approximately any PAM sequence.^[^ ^160^ ^]^

The discovery of new Cas nucleases requiring different and more relaxed PAM is another approach to expand the number of potential genomic target sites. Several Cas9 orthologs have been isolated, sharing the cleavage activity but differing in their PAM requirements. Of these, a new Cas9 nuclease identified from *Streptococcus canis* (ScCas9) is highly similar to SpCas9, sharing 89.2% sequence homology. However, it requires a less stringent PAM sequence of 5′‐NNG‐3′ compared to the 5′‐NGG‐3′ PAM required by SpCas9. SauriCas9 variant is another Cas9 ortholog that was identified in *Staphylococcus auricularis* (SauriCas9), recognizing a 5′‐NNGG‐3′ PAM sequence. In addition to this relaxed PAM, SauriCas9 exhibits a smaller size of 3.3 kb and high editing activity.^[^ ^161^ ^]^ Recently, a novel CRISPR/Cas9 system derived from *Lactobacillus rhamnosus* (LrCas9) was validated for efficient plant genome engineering, recognizing 5′‐NGAAA‐3′ PAM sequence.^[^ ^162^ ^]^ Many other Cas9 variants have been identified that could be useful for genome editing applications. However, they either require longer PAM sequences or exhibit lower overall activity levels than the widely used SpCas9 system.^[^ ^125^, ^163^, ^164^, ^165^ ^]^ We have summarized all the Cas proteins that have undergone trial experiments in **Table** **3**.

**Table 3:** Overview of Cas Nuclease Variants Utilized in CRISPR applications.

Classification	Nuclease	Origin	PAM Sequence (5′ to 3′)	Types of cells used for testing/trials	Protein size (aa)
**Class I** **type I‐E**	Cas3	*Escherichia coli, in silico *analysis of various prokaryotic genomes	No PAM sequence requirement	*Homo sapiens*	888
**Class II** **type II**	SpCas9	*Streptococcus pyogenes*	5′‐NGG‐3′	*Escherichia coli*,^[^ ^29^ ^]^ *Homo sapiens*,^[^ ^47^, ^324^ ^]^ bacteria,^[^ ^33^ ^]^ *Rattus norvegicus, Mus musculus*,^[^ ^325^, ^326^ ^]^ *Arabidopsis thaliana, Oryza sativa, Zea mays, Triticum aestivum, Gossypium hirsutum*.^[^ ^327^ ^]^	1,369
	Cas9‐NG	Engineered *Streptococcus pyogenes*	5′‐NG‐3′	*Homo sapiens* ^[^ ^328^ ^]^	1,368
	xCas9		5′‐NG‐3′ 5′‐GAA‐3′ 5′‐GAT‐3′	*Homo sapiens* ^[^ ^158^ ^]^	1,368
	VQR		5′‐NGA‐3′	*Danio rerio* and *Homo sapiens* ^[^ ^157^ ^]^	1,368
	VRER		5′‐NGCG‐3′	*Danio rerio* and *Homo sapiens* ^[^ ^157^ ^]^	1,368
	EQR		5′‐NGAG‐3′	*Danio rerio* and *Homo sapiens* ^[^ ^157^ ^]^	1,336
	SpCas9‐NRRH		5′‐NRRH‐3′	*Homo sapiens* ^[^ ^159^ ^]^	1,367
	SpCas9‐NRTH		5′‐NRTH‐3′	*Homo sapiens* ^[^ ^159^ ^]^	572
	SpCas9‐NRCH		5′‐NRCH‐3′	*Homo sapiens* ^[^ ^159^ ^]^	572
	SaCas9	*Staphylococcus aureus*	5′‐NGRRT‐3′ 5′‐NGRRN‐3′	*Homo sapien*s,^[^ ^157^ ^]^ *Mus musculus* ^[^ ^127^ ^]^	1,053
	St1Cas9	*Streptococcus thermophilus*	5′‐ NNAGAA‐3′	*Escherichia coli*,^[^ ^165^ ^]^ *Homo sapiens* ^[^ ^329^ ^]^	1,121
	NmeCas9	*Neisseria meningitidis*	5′‐NNNNGATT‐3′	*Homo sapiens* and *Streptococcus thermophilus* ^[^ ^157^, ^165^ ^]^	1,082
	Nme2Cas9	*Neisseria meningitidis*	5′‐NNNNCC‐3′	*Mus musculus* ^[^ ^163^ ^]^	1,082
	CjCas9	*Campylobacter jejuni*	5′‐NNNNRYAC‐3′	*Homo sapiens* and *Mus musculus* ^[^ ^164^ ^]^	984
	GeoCas9	*Geobacillus stearothermophilus*	5′‐NNNNCRAA‐3′	*Homo sapiens* ^[^ ^330^ ^]^	1,089
	LrCas9	*Lactobacillus rhamnosus*	5′‐NGAAA‐3′	*Triticum aestivum, Solanum lycopersicum, Larix laricina* ^[^ ^162^ ^]^	1,093
	CbCas9	*Chryseobacterium sp*.	5′ NNRAA 3′	*Escherichia coli* ^[^ ^331^ ^]^	1,442
**Class II** **type V**	LbCpf1 (Cas12a)	*Lachnospiraceae* bacterium	5′‐TTTV‐3′	*Homo sapiens*,^[^ ^55^ ^]^ *Oryza sativa*, *Gossypium hirsutum* ^[^ ^332^ ^]^	1,274
	AsCpf1 (Cas12a)	*Acidaminococcus* sp.	5′‐TTTV‐3′	*Homo sapiens*,^[^ ^55^ ^]^ *Shewanella oneidensis* ^[^ ^333^ ^]^	1,353
	FnCpf1 (Cas12a)	*Francisella novicida*	5′‐TTN‐3′	*Homo sapiens* ^[^ ^55^ ^]^	1,300
	MbCpf1 (Cas12a)	*Moraxella bovoculi 237*	5′‐TTN‐3′	*Homo sapiens*,^[^ ^55^ ^]^ *Gossypium hirsutum* ^[^ ^334^ ^]^	1,129
	AacCas12b (C2c1)	*Alicyclobacillus acidiphilus*	5′‐TTN‐3′	*Homo sapiens Mus musculus*,^[^ ^335^ ^]^ *Gossypium hirsutum* ^[^ ^336^ ^]^	1,129
	BhCas12b (C2c1)	*Bacillus hisashii*	5′‐ATTN‐3′, 5′‐TTTN‐3′ and 5′‐GTTN‐3′	*Homo sapiens*,^[^ ^337^ ^]^ *Shewanella oneidensis*,^[^ ^333^ ^]^ *Arabidopsis thaliana*,^[^ ^338^ ^]^ *Oryctolagus cuniculus* ^[^ ^339^ ^]^	1,108
	Cas14 (Cas12f)	*Uncultivated archaea*, (superphylum of extremophile archaea)	T‐rich PAM sequences, e.g. 5′‐TTTA‐3′ for dsDNA, no PAM sequence requirement for ssDNA	*Homo sapiens*,^[^ ^168^ ^]^ *Escherichia coli, Bacillus anthracis* ^[^ ^340^ ^]^	529

The emergence of type V variants has also expanded the targeting range of CRISPR nucleases. Cas12a (Cpf1) is a type V‐a variant, including AsCpf1 (*Acidaminococcus sp*. BV3L6), LbCpf1 (*Lachnospiraceae bacterium* ND2006), MbCpf1 (*Moraxella bovoculi* 237), and FnCpf1 (*Francisella novicida*), that recognizes short T‐rich PAM motifs. LbCpf1 and AsCpf1 strictly recognize the 5′‐TTTV‐3′ PAM motif, narrowing the number of available gRNAs compared to SpCas9. While MbCpf1 and FnCpf1 recognize 5′‐TTN‐3′ PAMs.^[^ ^55^ ^]^ The canonical PAM sequence of LbCpf1 and AsCpf1 has been further developed with relaxed specificity, shifting from 5′‐TTTV‐3′ to 5′‐TATV‐3′ and 5′‐TYCV‐3′ (Y = C or T).^[^ ^166^ ^]^ In addition to the Cas12a variants, the type V CRISPR system Cas12b (C2c1) is another promising tool for genome engineering, as it prefers AT‐rich PAM sequences.^[^ ^167^ ^]^ In addition to Cas12 variants, the CRISPR nuclease toolbox was expanded by reporting a new type V Cas14 nuclease from uncultivated archaea bacteria. This Cas14 nuclease displays unique capabilities – it targets single‐stranded DNA, does not require a PAM sequence for activation, and cleaves other single‐stranded DNA nonspecifically upon binding to the target sequence.^[^ ^168^ ^]^ Overall, the discovery and engineering of these Cas nucleases have expanded the targeting capabilities of CRISPR technology, providing more options for genome editing applications.

### 1.10 Risky Cleave‐Repair: The Limitations of the Traditional CRISPR Approaches

One major drawback of the traditional CRISPR/Cas9 gene editing technologies is the induction of DSBs in the DNA. This process is essential for gene editing, as the cell's natural DNA repair mechanisms are then activated to attempt to fix the break. However, the repair process is not always perfect. Recent research has shed additional light on the undesirable and previously unidentified byproducts that can arise from genome editing techniques relying on the induction of DSBs. These include large‐scale deletions in the genome, chromothripsis, and chromosomal rearrangements.^[^ ^169^, ^170^, ^171^, ^172^, ^173^ ^]^ Although strategies have emerged to enhance CRISPR‐mediated HDR, as highlighted above, precisely modifying genetic sequences to incorporate larger genetic elements remains inefficient, especially in cell types that lack efficient recombination machinery.^[^ ^174^ ^]^

The risks associated with DSBs have encouraged the development of alternative gene editing technologies, such as base editing (BE), prime editing (PE), and transposases, as well as programmable gene regulation.^[^ ^175^ ^]^ These innovative strategies address the limitations of CRISPR/Cas9‐induced DSBs by utilizing its programmable RNA‐guided capabilities to leverage fused effector domains to achieve targeted chemical modifications in the genome. This DSB‐independent editing approach can offer advantages over traditional CRISPR/Cas9 methods, such as reduced risk of unintended insertions, deletions, or chromosomal rearrangements.

### 1.11 Cleavage‐Free Editors: How Can the Destructive Nature of CRISPR‐mediated DSBs Be Overcome in Genome Editing?

#### 1.11.1 DNA Base Editing (BE)

BE is a groundbreaking genome engineering technology that has emerged as a powerful alternative to traditional CRISPR/Cas systems. Unlike CRISPR, base editors convert one DNA base to another without inducing DSBs, bypassing the need for homology‐directed repair. Due to its ability to easily introduce point mutations, BE has become highly popular and undergone rapid development since the initial base editors were first pronounced.^[^ ^176^ ^]^

**Figure 3:**
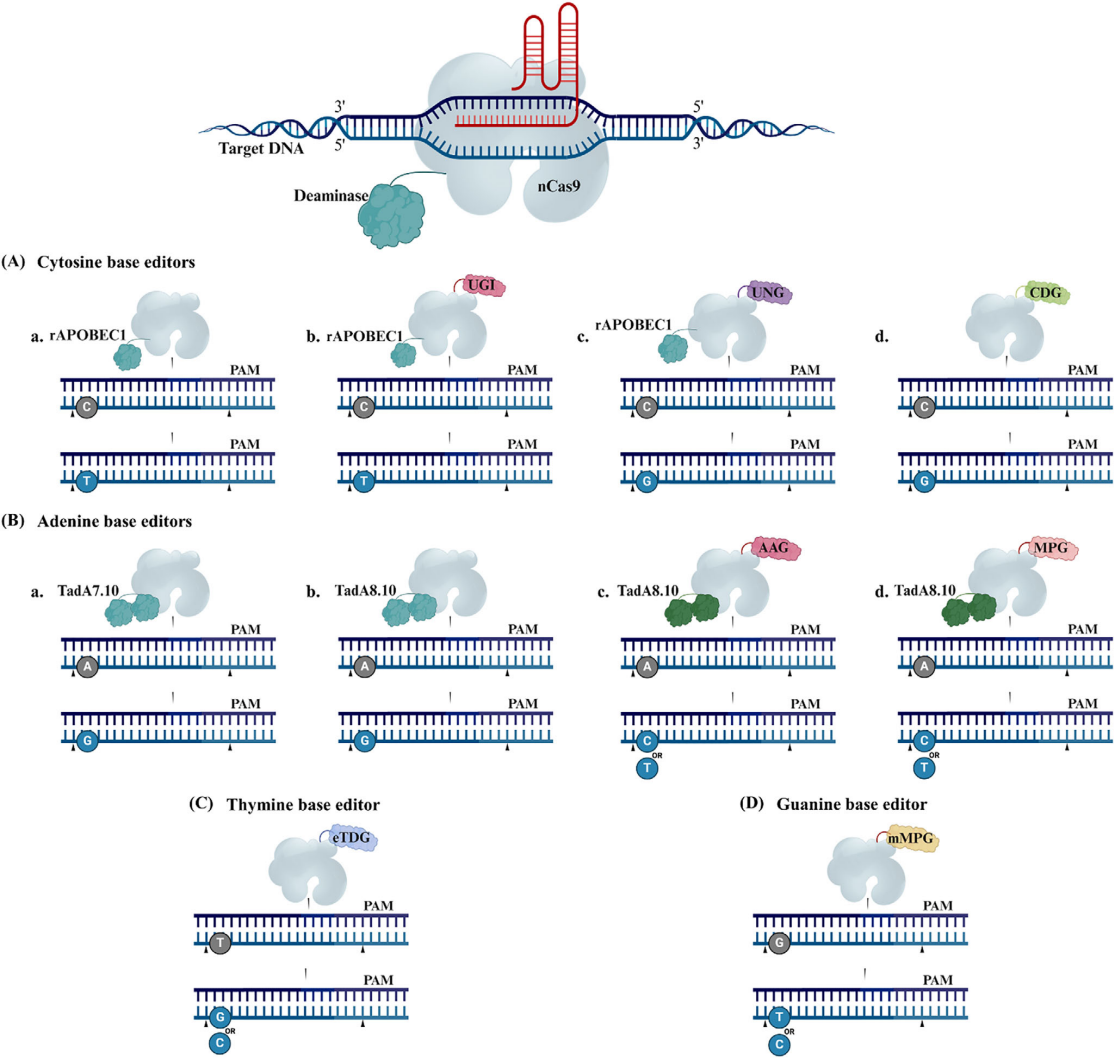
A schematic illustration of CRISPR base editors. An illustration depicting various base editors. A) Base editing (BE) includes cytosine, adenine, guanine, and thymine base editors. A) The cytosine base editing involves nCas9 protein fused to a) a cytidine deaminase enzyme, rAPOBEC1. This complex targets the DNA and converts cytosine (C) to uracil (U), which is read as thymine (T) during DNA replication, resulting in C‐to‐T substitutions. b) Combining an additional uracil glycosylase inhibitor (UGI) blocks the initial step of the base excision repair process, thereby favoring the retention of deaminated C as U. c) Conversely, when incorporating a uracil DNA N‐glycosylase (UNG) facilitates C‐to‐G transitions at the abasic sites. d) The CDG is directly fused to the nCas9‐mediated C‐to‐G conversion process, involving the specific binding of the CDG‐nCas9 complex to target genomic DNA loci. This complex directly excises cytosine or thymine, creating an apurinic site and enabling base editing. B) The adenine base editing utilizes nCas9 fused to a) an adenine deaminase enzyme derived from the tRNA deaminase TadA7.10. The ABE complex binds to the target DNA and converts adenine (A) to guanine (G), resulting in an A‐to‐G substitution. b) TadA8e is an enhanced version of TadA7.10 derived from phage‐assisted non‐continuous and continuous evolution, showing a preference for A‐to‐G conversion. c) Fusing the TadA8e to an alkyladenine DNA glycosylase (AAG) or d) an N‐methylpurine DNA glycosylase (MPG) permits A‐to‐C or A‐to‐T conversions. C) Thymine deaminase‐free base editor derived from engineered UNG attached directly to nCas9 without the need of any deaminase facilitates the substitution of T‐to‐G or T‐to‐C. D) Guanine deaminase‐free base editor derived from mutagenized MPG attached directly to nCas9 without the need of any deaminase induces the substitution of G‐to‐T or G‐to‐C.

The pioneering base editors, first reported in the scientific literature, were developed from the rat APOBEC1 cytosine deaminase (rAPOBEC1, CBE), with the initial version, called BE1, consisting of dCas9 linked to the active rAPOBEC1 enzyme guided by sgRNA, the dCas9 directs the deaminase to the target site, forming an R‐loop. Within this R‐loop, 5–8 nucleotides of single‐stranded target DNA become accessible to the deaminase, allowing the conversion of cytosine (C) to uracil (U), which is then recognized as thymine (T) by the cell, resulting in the C•G to T• adenine (A) conversion (**Figure** **3**Aa). This precise DSB‐independent mechanism makes BE particularly suitable for various organisms, including human,^[^ ^176^ ^]^ mouse,^[^ ^177^ ^]^ zebrafish,^[^ ^178^ ^]^ rabbit,^[^ ^179^ ^]^ monkeys,^[^ ^180^ ^]^ and plants (rice,^[^ ^181^ ^]^ cotton,^[^ ^182^ ^]^ soybean^[^ ^183^ ^]^).

Building upon the foundation of BE1, enhancements involved fusing a uracil glycosylase inhibitor (UGI) to the C‐terminus to prevent uracil excision, resulting in a more efficient BE2 variant. Further advancements led to the development of BE3, which integrates rAPOBEC1, UGI, and nCas9 (Figure 3Ab), inducing a single‐strand nick in the nonedited strand to promote efficient C•G to T•A conversions.^[^ ^176^ ^]^ Additionally, in the absence of UGI, cytosine deaminases can convert C•G through the base excision repair pathway.^[^ ^184^ ^]^ Thus, researchers fused rAPOBEC1 and nCas9 to a uracil DNA N‐glycosylase (UNG), which transforms U into an apyrimidinic/apurinic site (Figure 3Ac), facilitating improved C•G editing with a low occurrence of C•A editing.^[^ ^185^, ^186^ ^]^ Since then, the BE3 has become the most commonly used base editor. Progress in BE3 has expanded the molecular toolkit for genome engineering, allowing for various Cas9 variants and broadening the editing scope.^[^ ^187^ ^]^ Building on this progress, further optimizations led to the development of fourth‐generation base editors, BE4 and BE4‐Gam.^[^ ^188^ ^]^ Notably, existing base editors relying on deaminases have been shown to induce significant off‐target mutations in both cellular RNA and DNA independent of Cas9.^[^ ^189^, ^190^, ^191^, ^192^ ^]^ Recently, a deaminase‐free base editor for cytosine (DAF‐CBE) consists of a cytosine‐DNA glycosylase (CDG) variant that is tethered to an nCas9^[^ ^193^ ^]^ (Figure 3Ad).

Moreover, the evolution of the tRNA deaminase TadA produced a seventh‐generation adenine base editors (ABE7.8, 9, and 10) that can convert A•T to G•C. Remarkably, ABE7.10 has achieved efficient A•T to G•C conversions across diverse genomic loci (Figure 3Ba), surpassing BE3 in product purity.^[^ ^194^ ^]^ This innovation expands the scope of BE, enabling the programmable installation of all four conversions (A•G, C•T, G•A, and T•C). Despite this success, ABE7.10 showed limited deamination efficiency in certain plant genomes. To overcome this limitation, an enhanced version TadA8e (ABE8e) was developed, significantly boosting deamination activity^[^ ^195^ ^]^ (Figure 3Bb). To broaden the BE scope, the ABE8e system was harnessed to convert A•C and A•T when paired with alkyladenine DNA glycosylase (AAG)^[^ ^196^ ^]^ (Figure 3Bc) or N‐methylpurine DNA glycosylase (MPG)^[^ ^197^ ^]^ (Figure 3Bd). Additionally, rational design techniques were employed to engineer UNG, resulting in a deaminase‐free base editor capable of programmable T•G or T•C conversions with enhanced specificity toward T, known as enhanced thymine DNA glycosylase (eTDG), a thymine base editor^[^ ^193^, ^198^ ^]^ (Figure 3C). Another deaminase‐free base editor was developed as a guanine base editor, facilitating efficient G•T and G•C conversions^[^ ^199^ ^]^ (Figure 3D).

Overall, BE technologies address the limitations of CRISPR/Cas9 by providing a more precise, flexible, and versatile toolkit for genetic engineering. They reduce risks associated with traditional genome editing methods and broaden the possibilities for genetic modifications. The precision and flexibility of BE technologies facilitate the development of crop cultivars with enhanced traits, enabling them to better withstand biotic and abiotic stresses.^[^ ^200^, ^201^, ^202^ ^]^ In the medical field, BE's ability to make precise single‐base changes opens new avenues for gene therapies targeting genetic diseases in humans^[^ ^203^, ^204^, ^205^ ^]^ and mice,^[^ ^206^, ^207^ ^]^ offering significant advantages over traditional CRISPR tools.

#### 1.11.2 Prime Editing (PE)

**Figure 4:**
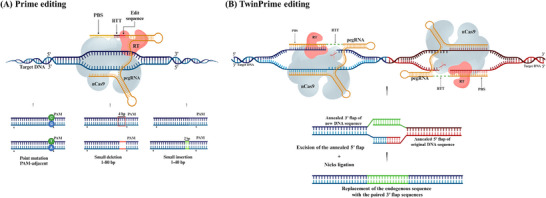
A schematic illustration of CRISPR prime editing. A) Prime editing uses a prime editing guide RNA (pegRNA) to direct the nCas9 protein that nicks one DNA strand and a reverse transcriptase enzyme that synthesizes the desired edit. This allows for a wide range of precise genetic modifications, including all base‐to‐base conversions, small insertions, and deletions. B) TwinPE system targets genomic DNA sequences with two protospacer sequences located on opposite strands. PE2–pegRNA complexes engage each protospacer, creating a single‐stranded nick and reverse transcribing the pegRNA‐encoded template with the desired insertion sequence. This process leads to the formation of 3′ DNA flaps, resulting in a hypothetical intermediate that has annealed 3′ flaps containing the edited sequence alongside 5′ flaps with the original DNA. The excision of the original sequence from the 5′ flaps, followed by the ligation of the 3′ flaps at the corresponding excision sites, produces the intended edited product. Abbreviations: PBS, prime binding site; RT, reverse transcriptase; RTT, reverse transcriptase template.

PE is a recently developed technology that can precisely introduce a wide variety of genetic changes, including all 12 possible types of single nucleotide substitutions, as well as small insertions and deletions.^[^ ^208^ ^]^ The core PE system comprises an nCas9, a specialized PE guide RNA (pegRNA), and a modified reverse transcriptase enzyme. The nCas9 creates a single‐strand nick in the DNA. The pegRNA then hybridizes to the exposed 3′ end, providing a primer‐template complex. The reverse transcriptase domain then copies this template into the target DNA strand, synthesizes the new DNA sequence, and inserts it into the target locus. This avoids the need for donor DNA templates or the generation of DSBs. Cellular DNA repair processes then excise the unedited 5′ flap and incorporate the edited 3′ flap, generating a heteroduplex DNA molecule (**Figure** **4**A). An additional nicking step is typically performed to fully install the edit, using a sgRNA to nick the unedited strand of the heteroduplex. This stimulates DNA repair to use the edited strand as a template, synthesizing a new complementary strand to replace the original unedited sequence, resulting in a fully edited DNA duplex.^[^ ^208^ ^]^

PE offers greater targeting flexibility compared to traditional CRISPR‐based genome editing approaches. They can install nucleotide edits at sites located over 30 bp away from the nCas9 cleavage site, making prime editors less dependent on the availability of PAM sequences, an important constraint for Cas9‐based nucleases.^[^ ^209^ ^]^ Prime editors also showed lower rates of off‐target editing compared to Cas9 nucleases,^[^ ^208^ ^]^ likely because they require three hybridization steps after the initial protospacer‐spacer interaction, which helps avoid off‐target sites. However, more research is needed to fully characterize potential unintended effects, such as off‐target incorporation of the reverse‐transcribed template.

Early evaluation revealed limitations such as inefficient processing, poor thermal stability, and low affinity of the reverse transcriptase component.^[^ ^210^ ^]^ To optimize PE, researchers have focused on increasing the local concentration of PE components,^[^ ^211^ ^]^ engineering stable and effective pegRNA designs,^[^ ^212^, ^213^, ^214^ ^]^ and incorporating nuclear localization signals for better delivery.^[^ ^212^, ^213^ ^]^ Overcoming PAM sequence restrictions with variants like PE2‐SpG^[^ ^215^ ^]^ and PE2‐NG^[^ ^216^ ^]^ has also broadened PE's applicability. Most recently, an advanced PE tool known as EXPERS has been developed, allowing for efficient editing on both sides of the pegRNA nick.^[^ ^217^ ^]^ Nonetheless, PE still faces certain challenges, particularly in achieving high‐efficiency insertion of long DNA fragments, suggesting opportunities to combine and synergize different enhancements for greater impact.

A recent approach, namely TwinPE enhances PE capabilities for larger gene insertions by using paired pegRNAs targeting opposing DNA strands with complementary editing templates at the 3′ flap.^[^ ^218^ ^]^ This method allows for precise insertion of new DNA sequences by creating and hybridizing 3′ complementary overhangs, leading to efficient replacement of the original DNA (Figure 4B). TwinPE demonstrates greater precision and efficiency in large sequence modifications compared to previous systems.

#### 1.11.3 CRISPR‐Associated Transposases/ Recombinases

Transposons are DNA sequences that can move or “transpose” within the genome, facilitated by transposases, which recognize transposon sequences and catalyze their insertion into target sites. Natural transposase can be modified for inserting exogenous genes into genomes,^[^ ^219^ ^]^ offering several advantages over traditional gene knock‐in tools. These include a lower risk of off‐target effects, the ability to carry larger DNA fragments (over 100 000 bp), and higher activity levels, making transposase‐based systems promising for gene insertion where donor DNA size is critical.

**Figure 5:**
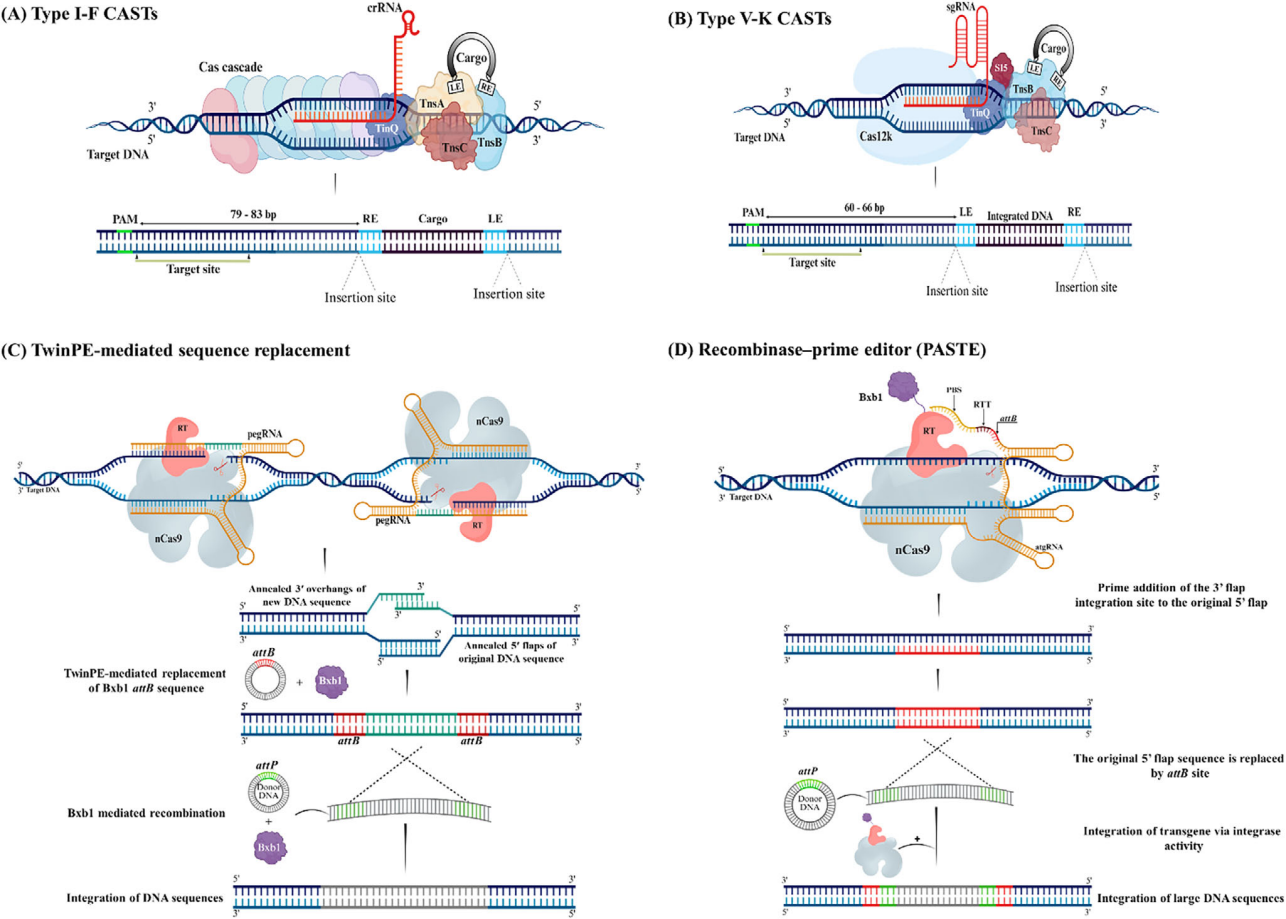
Overview of CRISPR/Cas combines transposases/recombinases for long sequence insertion. Type I‐F CRISPR‐associated transposase (CAST) and type V‐K CAST can facilitate the insertion of cargos. A) Type I‐F CAST binds to a target in an RNA‐guided manner, aided by bacterial proteins TniQ and TnsA‐C, along with the expression of CASCADE (Cas) proteins; Cas6, Cas7, and Cas8‐5; which help disassemble the post‐transposition complex. B) In contrast, type V‐K CAST also binds to a target in an RNA‐guided manner but requires the coexpression of TniQ and a bacterial S15 protein. Both CAST systems introduce target site duplications and leave scars from left end (LE) and right end (RE) of the cargo. This prime editing‐based insertion strategy relies on integrase or recombinase‐catalyzed donor insertion. C) Similar to the TwinPE system, *attB* and/or *attP* sites can be integrated at the edited site to enable subsequent insertion of large DNA sequences. This is achieved by delivering a plasmid containing the large DNA sequence flanked by *attP* sites, along with a coding sequence for the serine recombinase Bxb1, which catalyzes the integration of the DNA cargo. D) Programmable addition through site‐specific targeting element (PASTE) is designed for targeted insertion. An attachment site‐containing guide RNA (atgRNA) directs a PASTE fusion complex; comprising Cas9 nickase (nCas9), reverse transcriptase (RT), and integrase; to a specific genomic locus, facilitating the integration of an *attB* site. The integrase then recognizes the attB site and integrates a large DNA fragment flanked by *attP* sites without creating a double‐stranded break (DSB).

The development of gene‐insertion tools has shifted toward combining transposase‐based systems with CRISPR/Cas technology, addressing traditional transposon systems' limitations, such as poor targeting specificity and limited programmability. To date, two main subtypes of CRISPR‐associated transposon (CAST) systems have been characterized for RNA‐guided targeting of Tn7‐like transposons. The I‐F CAST systems employ Cascade complexes that lack the Cas3 nuclease component^[^ ^220^ ^]^ (**Figure** **5**A), while the V‐K CAST systems use Cas12k effectors with naturally inactive nuclease domains^[^ ^221^ ^]^ (Figure 5B). In both systems, researchers demonstrated the ability to integrate genomic cargo into *E. coli* genome. This approach has been successfully applied in human cells,^[^ ^222^ ^]^ opening new avenues for eukaryotic genome engineering. The discovery of a fully programmable RNA‐guided system allows for genomic alterations without the need for DSBs or HDR. More details regarding CASTs are well described by Anzalone et al. and Liu et al.^[^ ^223^, ^224^ ^]^

CRISPR effectors can be programmed to bind to specific DNA sequences, and engineered systems now exploit this feature to directly recruit recombinases. Recent advances in CASTs have encouraged the development of recombinases for targeted genetic engineering. Despite efficiency and sequence constraints, engineered Cas‐fused recombinases can manipulate plasmid substrates and eliminate targeted genomic loci in human cells.^[^ ^225^ ^]^ To address sequence limitations, nonprogrammable recombinases targeting specific genetic sequences have been developed that can delete specific genetic sequences.^[^ ^226^ ^]^ Additionally, to overcome the high sequence constraints, a recombinase domain dimer (Ginβ) was fused to dCas9, using two sgRNAs to direct each Ginβ monomer to distinct dCas9 units at the site of interest. This complex displayed low efficiency in mediating large deletions in mammalian genomes, yet moderate efficacy on plasmid substrates.^[^ ^225^ ^]^ Despite these limitations, the recombinases' ability to perform diverse genome‐modifying activities makes them promising targets for constant development.

Recently, TwinPE was integrated with a serine recombinase that enables site‐specific integration and inversion of large DNA sequences^[^ ^218^ ^]^ (Figure 5C). Similarly, integrating reverse transcriptases (RT) and large serine integrases with Cas9 enables programmable incorporation of up to ≈36 kb at once.^[^ ^227^ ^]^ This strategy involves the combination of PE and site‐specific serine integrase known as programmable addition via site‐specific targeting element (PASTE) (Figure 5D). While these improvements have steadily increased editing efficiency and flexibility in mammals,^[^ ^204^, ^208^, ^228^ ^]^ progress in plant cells remains limited. Among several successful prime editing examples,^[^ ^229^, ^230^, ^231^ ^]^ the PrimeRoot method stands out for enabling targeted large DNA insertions in plants, achieving up to ∼11 kb.^[^ ^232^ ^]^

#### 1.11.4 Programmable Gene Regulation

CRISPR‐mediated transcriptional modulation involves the manipulation of CRISPR/Cas system to temporarily activate (CRISPRa) or interfere (CRISPRi) gene expression without altering the DNA sequence. This typically employs catalytically inactive Cas proteins, such as dCas9, which bind to specific DNA sequences without losing nuclease activity, thereby preventing DNA cleavage. These systems can modulate transcription through recruiting transcriptional activators or repressors to target gene loci.

**Figure 6:**
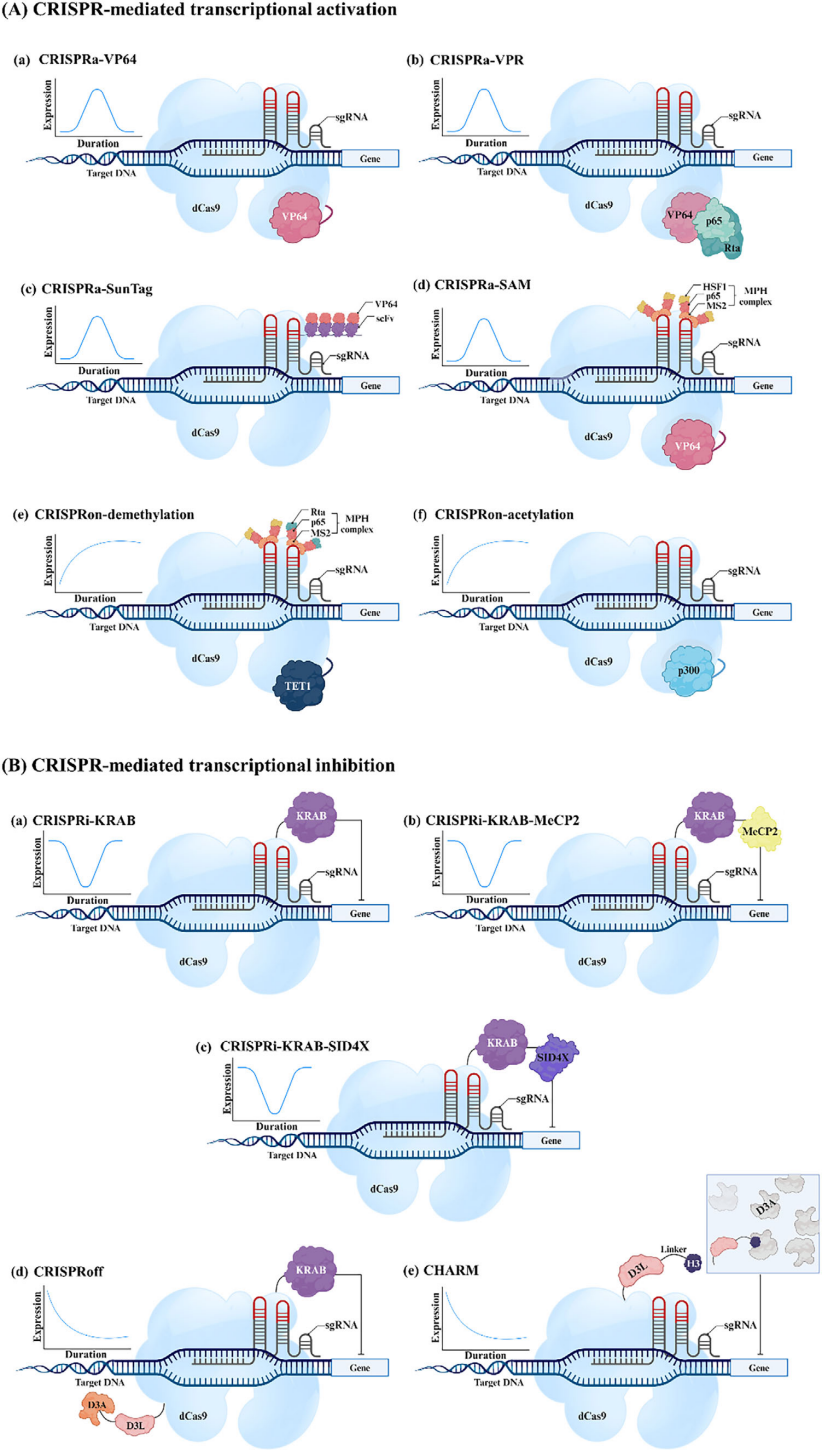
CRISPR‐mediated transcriptional modifications. (A) CRISPR‐mediated transcriptional activation (CRISPRa). a) The CRISPRa‐VP64 system combines dead Cas9 (dCas9) and a guide RNA (gRNA) scaffold with the VP64 transcriptional activator to recruit the transcriptional activation domain to the target gene. b) For robust transcriptional activation, three strong activation domains—VP64, p65, and Rta—are fused to dCas9, resulting in a higher transcriptional output compared to VP64 alone. c) The SunTag system uses a repeating peptide array (SunTag) fused to dCas9, which recruits multiple copies of an antibody‐fused activation domain (such as VP64). Each peptide in the array can bind an activator, thus amplifying the recruitment of transcriptional activators to the target gene. d) SAM (Synergistic Activation Mediator) combines dCas9 with a modified gRNA scaffold that recruits additional activation domains, MS2, p65, and HSF1. e) CRISPRon involves the fusion of dCas9 with a modified gRNA scaffold that recruits Rta, p65, and MS2, which together bind to TET1. The dCas9‐TET1 complex binds to the target gene, where TET1 induces DNA demethylation, leading to transcriptional activation. f) CRISPRon involves the induction of acetylation and gene expression through the fusion of dCas9 with the catalytic core of the human acetyltransferase p300. This fusion facilitates the acetylation of histone H3 at lysine 27 in its target regions, resulting in strong transcriptional activation of target genes from their respective promoters. B) CRISPR‐mediated transcriptional inhibition (CRISPRi). a) The dCas9‐KRAB complex binds to the target gene, recruiting the repressive chromatin modifier (Krüppel‐associated box) to silence gene expression. b) The dCas9‐MeCP2 complex binds to the target gene, utilizing MeCP2's repressive function to inhibit gene transcription. c) The dCas9‐SID4X complex recruits additional repressive complexes to the target gene. d) CRISPRoff involves the binding of the dCas9‐KRAB fusion to the DNA methyltransferases Dnmt3A and Dnmt3L. DNA methyltransferase domains facilitate the methylation of CpG sites in the target genomic region, resulting in stable transcriptional repression. e) The CHARM system utilizes a coupled histone tail (H3‐tail) and the Dnmt3L protein to modulate the autoinhibition of methyltransferases. When the H3‐tail binds to Dnmt3L. It triggers a conformational change that releases the autoinhibitory domain of the methyltransferase, leading to its activation that facilitates endogenous DNA methylation and gene silencing.

Early CRISPRa systems involved fusing a single transcriptional activator, VP64, to dCas9 to activate targeted gene expression^[^ ^233^ ^]^ (**Figure** **6**Aa). Later systems combined dCas9 with three^[^ ^234^, ^235^ ^]^ (Figure 6Ab), or multiple activation domains, such as the dCas9‐SunTag system (Figure 6Ac), which amplifies the activation signal,^[^ ^236^ ^]^ and the SAM (Synergistic Activation Mediator) system (Figure 6Ad), which recruits multiple transcriptional activators for robust gene activation.^[^ ^237^ ^]^ Despite ongoing developments, the mechanisms of various activators across different cell types and genomic targets are still not fully understood, impeding the creation of universally effective technologies.^[^ ^238^ ^]^ Several attempts have been made to identify new transcription activator domains with varying levels of activity strength.^[^ ^239^, ^240^, ^241^ ^]^ By further optimization, the CRISPRa system was developed to stably enable customizable transcriptome modification (CRISPRon) by coupling CRISPRa with epigenetic modifiers, such as histone acetyltransferases^[^ ^242^ ^]^ (Figure 6Ae), and TET1 DNA demethylase^[^ ^243^ ^]^ (Figure 6Af), to achieve gene activation.

For gene repression, CRISPRi incorporates the KRAB (Krüppel‐associated box, a zinc finger protein 10) chromatin repressor domain into the dCas9,^[^ ^244^ ^]^ which alters chromatin to a transiently repressive state, thereby inhibiting target gene transcription (Figure 6Ba). Subsequent optimizations have included substituting KRAB with more powerful zinc finger imprinted 3 (ZIM3)‐KRAB domain to enhance efficiency.^[^ ^245^ ^]^ Further improvements included fusing dCas9‐KRAB with additional repressor domains like SID4X^[^ ^246^ ^]^ (Figure 6Bb) or MeCP2^[^ ^247^ ^]^ (Figure 6Bc) to augment transcriptional inhibition. However, this strategy led to higher off‐target effects and cellular toxicity in K562 cell lines.^[^ ^248^ ^]^ With further refinement, CRISPRi was developed into CRISPRoff for programmable transcriptome modification. This system achieves stable transcriptional repression by fusing dCas9‐KRAB to DNA methyltransferase domains, Dnmt3A^[^ ^249^ ^]^ and Dnmt3L,^[^ ^250^ ^]^ demonstrating minimal off‐target activity independent of sgRNA (Figure 6Bd). However, its inherent complexity poses delivery and toxicity challenges that impede its therapeutic application. To solve delivery and toxicity issues, researchers have introduced an innovative approach known as the Coupled Histone tail (H3‐tail‐Dnmt3L) for Autoinhibition Release of Methyltransferase (CHARM). This advanced technology successfully recruits and activates endogenous DNA methyltransferases (Figure 6Be), minimizing transgene size and cytotoxicity for enhanced therapeutic viability.^[^ ^251^ ^]^

**Figure 7:**
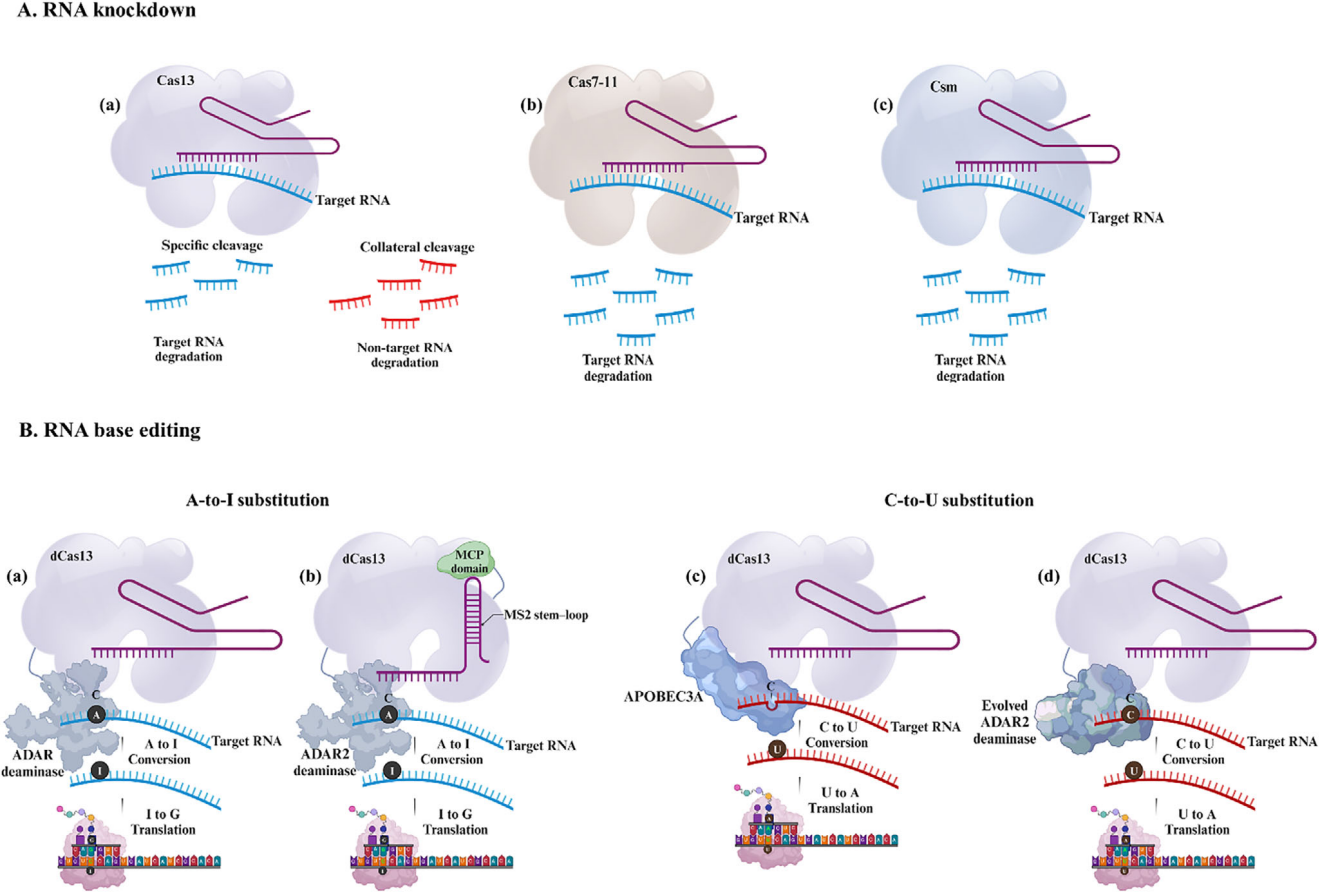
CRISPR‐mediated RNA modifications. This figure illustrates the process of RNA modifications using CRISPR technology. A) RNA Knockdown via Cas13, Cas7‐11, and Csm. a) The Cas13 system is a CRISPR‐associated, RNA‐guided nuclease that targets and cleaves single‐stranded RNA. A distinctive feature of Cas13 is collateral cleavage, which results in the degradation of non‐target RNAs. b) In contrast, Cas7–11 is a large single‐protein effector that enables RNA‐guided RNA degradation with reduced cellular toxicity and no collateral activity. c) CRISPR–Csm complex is a multisubunit structure that also utilizes a programmable RNA‐guided mechanism to degrade target RNAs without causing collateral damage. B) Precise nucleotide editing of RNA is achieved through deaminase‐mediated conversion of adenosine to inosine (A‐to‐I) and cytidine to uridine (C‐to‐U). a,b) RNA A‐to‐I base editing relies on ADAR (adenosine deaminases acting on RNA) enzymes to target double‐stranded RNA (dsRNA) substrates. ADAR deaminases can be directed to specific sites using catalytically inactive Cas13 (dCas13). Alternatively, guide RNAs can be fused to the bacteriophage MS2 coat protein (MCP). The MCP attaches to the MS2 stem‐loop RNA, and the MS2 loop region subsequently recruits the MCP‐ADAR2 to the editing site. c,d) RNA C‐to‐U base editing involves two main approaches: RESCUE and CURE. In the RESCUE approach, an artificially evolved ADAR2 is used to deaminate cytosine, recruited to target sites via dCas13. In the CURE approach, dCas13 mediates the recruitment of the APOBEC3A mutant to deaminate RNA within a small loop substrate.

Beyond genome editing tools, RNA‐guided CRISPR nucleases such as Cas13, Cas7‐11, and Csm have emerged as alternative tools to target RNA, enabling diverse transcriptome engineering applications without inducing DSBs. Cas13a, from the class 2 type VI CRISPR system, degrades target RNA molecules using programmable gRNAs, thereby attenuating the expression of the targeted gene, while also exhibiting collateral cleavage activity for RNA sequences (*in cis*) complementary.^[^ ^252^ ^]^ In addition to degrading bound RNA targets, Cas13a extensively cleaves adjacent single‐stranded RNA (ssRNA), leading to *trans* RNA degradation, also known as collateral degradation (**Figure** **7**Aa).^[^ ^253^ ^]^ Notably, this activity is a cell type‐dependent, which could pose a challenge to CRISPR/Cas13a specificity and in vivo application. Recent innovations have fused dCas13 with methyltransferases and demethylases to regulate m^6^A modifications at specific RNA sites.^[^ ^254^ ^]^ New Cas13 orthologues have also been discovered from *Prevotella* sp. (PspCas13b),^[^ ^255^ ^]^ *Porphyromonas gulae* (PguCas13b),^[^ ^256^ ^]^ and *Ruminococcus flavefaciens* (RfxCas13d),^[^ ^257^ ^]^ showing improved knockdown efficiency but with collateral activity. To address this, the RfxCas13d variant has been engineered to eliminate unselective RNA binding and reduce collateral activity, achieving undetectable collateral cleavage *in trans* RNA.^[^ ^257^ ^]^ Moreover, the use of Cas13X has also shown high knockdown efficiency with no collateral cleavage activity.^[^ ^258^ ^]^ Additional approaches to enhance Cas13‐mediated RNA knockdown involve chemical modifications of RNA bases,^[^ ^259^ ^]^ large‐scale screenings to determine gRNA design principles,^[^ ^260^ ^]^ and gRNAs computational predictions.^[^ ^261^ ^]^ These discoveries enable applications in programmable RNA targeting in both eukaryotic and prokaryotic cells.

Cas7–11, a class 1 type III‐E CRISPR system, is another single RNA‐targeting nuclease that cleaves target RNA for gene knockdown^[^ ^17^ ^]^ without collateral activity. Unlike Cas13, Cas7–11 lacks collateral cleavage activity, providing higher specificity and enabling the possibility of in vivo applications (Figure 7Ab).^[^ ^262^ ^]^ This was achieved by truncating the Cas7‐11 protein to fit AAV packaging and delivery, overcoming Cas13 drawbacks.^[^ ^263^ ^]^ The broader CRISPR/Cas Type III system, particularly the Csm complex, also offers effective RNA targeting for transcriptome engineering, showing high knockdown efficiency and minimal off‐target activity.^[^ ^264^, ^265^ ^]^ It consists of multiple subunits, including Csm proteins (Csm1 to Csm5), and a programmable complementary gRNA to the target RNA (Figure 7Ac). Together, Cas13, Cas7‐11, and Csm represent powerful tools for RNA‐based transcriptome engineering, allowing precise control over gene expression without altering the underlying DNA sequence.

#### 1.11.5 RNA Base Editing

RNA BE is a technology that allows for precise and targeted modifications of RNA molecules without manipulating the underlying DNA sequence. This approach harnesses the specificity of CRISPR systems combined with RNA‐modifying enzymes to achieve specific nucleotide changes in RNA transcripts. Like DNA‐BE, the RNA‐BE system uses the catalytically inactive version of RNA‐targeting Cas13 (dCas13), guided by programable gRNA, to direct the editing machinery to the specific RNA sequence of interest. These proteins are fused with adenosine deaminase acting on RNA (ADAR), to mediate the editing process. The ADAR enzyme converts adenosine (A) to inosine (I) in RNA, which is read as guanosine (G) during translation (Figure 7Ba). This allows for precise RNA‐BE, reaching up to 30% RNA editing efficiency; however, it shows off‐target edits. To address the off‐target editing, the dCas13‐ADAR was engineered to develop dCas13‐ADAR2,^[^ ^256^ ^]^ which significantly reduced off‐target effects (Figure 7Bb).

Leveraging the dCas13‐ADAR2, CRISPR‐mediated RNA editing has been upgraded to include a cytosine deaminase called RNA Editing for Specific C‐to‐U Exchange (RESCUE), achieving C‐to‐U conversion.^[^ ^266^ ^]^ This process involves mutating ADAR2 to minimize off‐target effects. Despite this modification, RESCUE still catalyzes A‐to‐I deamination (Figure 7Bc). Alternatively, a new cytosine deaminase RNA editor, CURE, was developed by engineering the cytosine deaminase rAPOBEC3A and bonding it to dCas13.^[^ ^267^ ^]^ This editor works in combination with gRNA to create a flexible loop at target sites (Figure 7Bd). Further advancements introduced a smaller and more efficient RNA base editor. This was achieved by fusing RNA‐guided EcCas6e nuclease to ADAR deaminases, resulting in compact and efficient RNA base editors (ceRBEs), which demonstrate elevated A‐to‐I and C‐to‐U conversion rates with minimal transcriptome off‐target edits compared to other RNA editors.^[^ ^268^ ^]^ Additionally, further cytosine deaminase RNA editors have been developed^[^ ^269^ ^]^ and well‐reviewed in.^[^ ^270^, ^271^ ^]^

### 1.12 Conclusion and Future Perspectives

**Figure 8:**
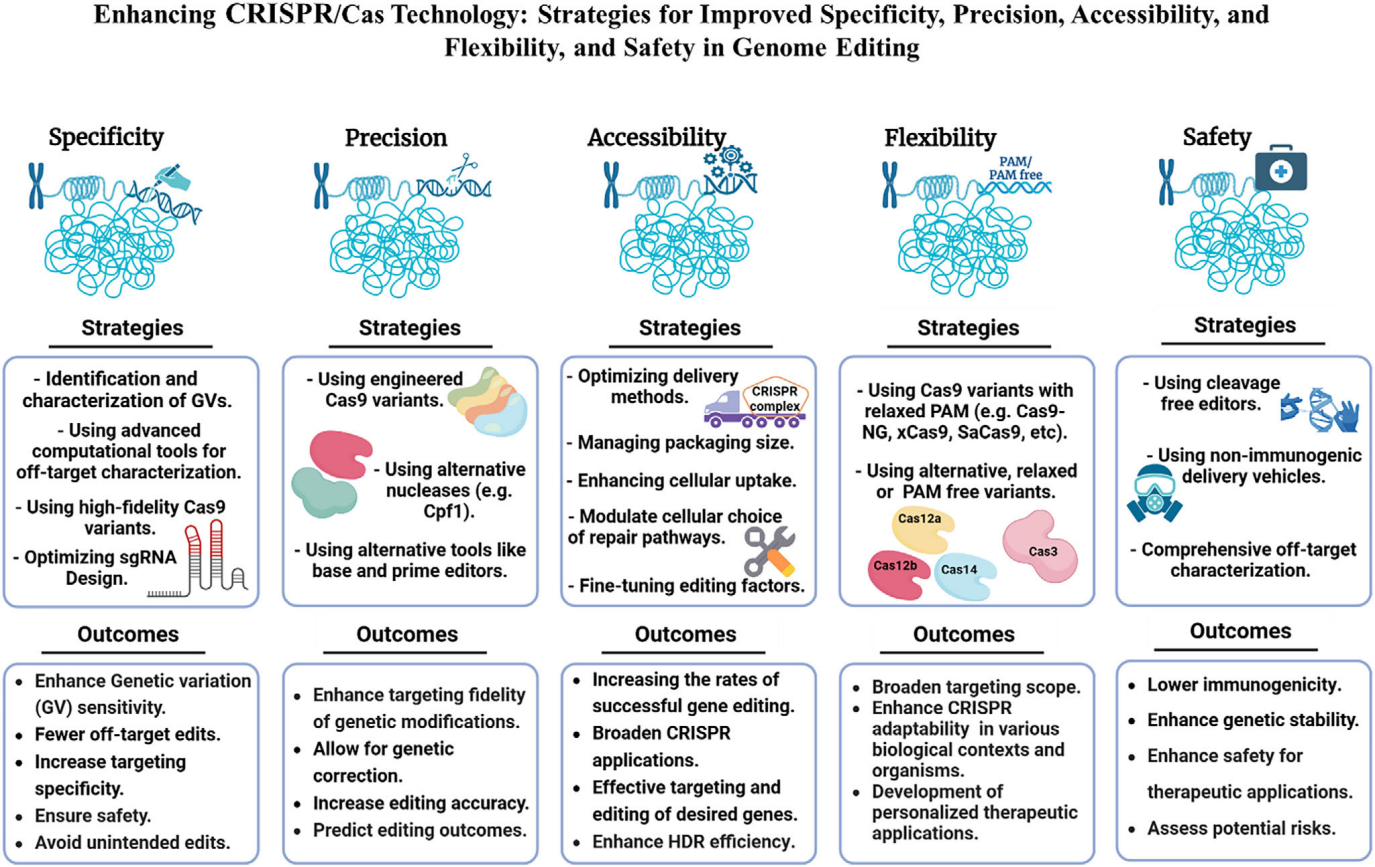
Schematic illustration of strategies for enhancing CRISPR/Cas technology in genome editing, focusing on specificity, precision, accessibility, flexibility, and safety. To enhance CRISPR specificity, strategies include the identification and characterization of genetic variants (GVs), the use of advanced computational tools for off‐target analysis, high‐fidelity Cas9 variants, and optimized sgRNA design can result in enhancing GV sensitivity, and reducing off‐target edits that increase targeting specificity, ensure safety, and avoid unintended modifications. To improve precision, employing engineered Cas9 variants, alternative nucleases (e.g., Cpf1), and advanced tools like base and prime editors can enhance targeting fidelity, allow genetic corrections, increase editing accuracy, and predict editing outcomes. For accessibility, we highlight approaches including optimizing delivery methods, managing packaging size, enhancing cellular uptake, modulating repair pathway choices, and fine‐tuning editing factors. These strategies are designed to improve delivery efficiency, bias the cellular repair pathway toward desired outcomes, and enhance the efficiency of Homology Directed Repair (HDR). Additionally, the CRISPR complexes are illustrated alongside strategies that utilize Cas9 variants with relaxed or PAM‐free options to expand targeting capabilities. Key approaches include using diverse Cas variants (e.g., Cas12b, Cas3) to broaden the targeting scope and enhance adaptability across different biological contexts and organisms. The safety aspect emphasizes the use of non‐immunogenic delivery vehicles, comprehensive off‐target characterization, and the development of therapeutic applications while addressing potential risks. This comprehensive approach aims to enhance the efficacy and safety of CRISPR‐based interventions.

The CRISPR/Cas9 system has undeniably transformed genetic engineering, yet its full potential remains constrained by persistent challenges in specificity, precision, accessibility, flexibility, and safety. While incremental progress has been made, the next era of CRISPR innovation demands paradigm‐shifting solutions that transcend incremental improvements. Addressing these limitations is paramount for its successful integration into various sectors, including agriculture, medicine, and biotechnology. As highlighted in our review, ongoing advancements are tackling these challenges through targeted technological solutions (**Figure** **8**).

Off‐target effects remain the Achilles’ heel of CRISPR therapies, but emerging solutions now extend beyond high‐fidelity Cas9 variants (e.g., SpCas9‐HF1, HypaCas9). Recent structural insights into Cas9's DNA recognition mechanism have enabled rational engineering of ultra‐precise variants like Sniper‐Cas9, which balances specificity with retained on‐target efficiency. However, even these advances cannot fully eliminate mosaicism in vivo, particularly in postmitotic tissues. Future efforts must integrate predictive and corrective approaches. For prediction, machine learning frameworks (e.g., DeepCRISPR, Elevation) trained on multiomic datasets (epigenetic states, chromatin accessibility) could dynamically predict cell type‐specific off‐target risks, enabling patient‐specific editing regimens. For correction, self‐inactivating “hit‐and‐run” systems, such as Cas9 ribonucleoprotein (RNP) complexes with transient activity or light‐inducible degradation tags, could minimize prolonged exposure and collateral damage.

On the other hand, prime editing and base editing represent monumental leaps toward precision, yet their reliance on bulky reverse transcriptase domains limits delivery efficiency. A promising frontier lies in miniaturized editors: for example, fusing compact deaminases (e.g., TadA‐8e) with nCas9 to create constructs compatible with AAV delivery. Parallel advances in epitope masking to evade preexisting immunity against bacterial Cas proteins will be critical for clinical translation.

A cornerstone of CRISPR advancement lies in refining delivery systems to enhance accessibility, efficiency, and specificity. While viral vectors offer high delivery efficiency, their restricted cargo capacity hinders their use for larger editors, such as prime editing systems. Conversely, nonviral platforms, such as LNPs, face challenges with transient expression and inefficient endosomal escape, undermining their utility for durable edits. To improve accessibility, engineered AAV capsids (e.g., AAV‐SynPrime) now enable tissue‐specific targeting of challenging sites like the blood‐brain barrier, while hybrid viral‐LNP systems expand cargo capacity for kilobase‐scale integrations. Simultaneously, compact Cas variants (e.g., Cas12f) bypass viral packaging limits, broadening delivery options for bulky tools. Enhancing efficiency demands innovations like LNPs functionalized with pH‐sensitive peptides, which achieve >90% endosomal escape, resolving a critical barrier, and ultrasound‐triggered lipid bubbles that enable spatiotemporal control with >60% editing rates in preclinical models. Meanwhile, ensuring specificity requires addressing preexisting immunity to synthetic AAVs and optimizing scalability in complex tissues. Future strategies, such as machine learning‐guided capsid design and biomimetic coatings mimicking endogenous exosomes, aim to refine targeting precision and durability. By synergizing viral precision with nonviral adaptability, next‐generation delivery systems could democratize CRISPR access in previously inaccessible tissues, accelerating therapies for neurological disorders and enabling climate‐resilient crops through precise, large‐scale genomic integrations.

The advent of DSB‐free editing tools, including base editors and prime editors, which transcend simple knockouts to enable multiomic reprogramming of genomes, epigenomes, and transcriptomes, while significantly reducing the risks associated with DSBs in traditional Cas9‐mediated editing. Scaling these systems to enable large‐scale insertions (>10 kb) or multiplex editing, through integration with recombinases, twin prime editing, or CASTs, holds transformative potential for both therapeutic and agricultural applications. For instance, CASTs could facilitate the precise integration of entire metabolic pathways (e.g., nitrogen fixation), bypassing the reliance on error‐prone HDR in nondividing cells. In parallel, RNA‐targeting platforms, such as Cas13‐ADAR fusions, offer transient and reversible transcriptome modulation, providing a safer alternative. Beyond sequence editing, CRISPR's expansion into multiomics regulation is critical: epigenetic engineering via dCas9‐effector fusions (e.g., p300 acetyltransferase or DNMT3A methyltransferase) enables locus‐specific chromatin remodeling, though sustaining these modifications in vivo necessitates integration with synthetic feedback circuits to enhance persistence. However, challenges remain, including the ≈5–10% off‐target integration rates of CASTs and the limited efficiency of RNA editors in low‐abundance transcripts. Advancing these technologies hinges on ultrahigh‐fidelity Cas variants with expanded PAM compatibility (e.g., SpRY) and modular architectures that integrate editing, epigenomic regulation, and transcriptional control. By converging these innovations, CRISPR technologies could usher in an era of precision genome engineering, enabling curative therapies for genetic disorders, climate‐resilient crops, and programmable control over cellular function.

Artificial Intelligence (AI) plays a crucial role in refining the specificity, precision, and accessibility of CRISPR technology. Machine learning algorithms can analyze vast genomic datasets to predict off‐target sites and optimize sgRNA design, significantly enhancing the accuracy of genome editing. AI and machine learning could also optimize sgRNA design, predict repair outcomes, and model complex biological interactions, accelerating discovery. Additionally, AI can assist in the identification of novel Cas9 variants and their interactions with different PAM sequences, accelerating the discovery of tools that expand the targeting range of CRISPR systems.

As CRISPR technology advances, ethical considerations surrounding its applications become increasingly pertinent. It is essential to develop comprehensive regulatory frameworks that govern the use of genome editing technologies. Future perspectives should emphasize establishing guidelines that ensure the safety and ethical implications of CRISPR applications, particularly in human germline editing and agricultural biotechnology.

In summary, the recent advancements in CRISPR/Cas9 technology underscore its potential to revolutionize genetic engineering. By addressing challenges related to off‐target effects, PAM restrictions, and delivery mechanisms, we can enhance specificity, precision, flexibility, accessibility, and safety. The integration of alternative genome editing technologies, coupled with responsible ethical considerations, will be pivotal in shaping the future landscape of genome editing. Through collaborative efforts and the strategic application of AI, we can harness the power of CRISPR technology to develop sustainable solutions for pressing global challenges, ultimately improving human health, food security, and environmental sustainability.

## Conflict of Interest

The authors declare no conflict of interest.
